# Branched Methoxydiphenylamine-Substituted Carbazole Derivatives for Efficient Perovskite Solar Cells: Bigger Is Not Always Better

**DOI:** 10.1021/acs.chemmater.1c02114

**Published:** 2021-08-19

**Authors:** Povilas Luizys, Jianxing Xia, Maryte Daskeviciene, Kristina Kantminiene, Ernestas Kasparavicius, Hiroyuki Kanda, Yi Zhang, Vygintas Jankauskas, Kasparas Rakstys, Vytautas Getautis, Mohammad Khaja Nazeeruddin

**Affiliations:** † Department of Organic Chemistry, Kaunas University of Technology, Radvilenu pl. 19, Kaunas 50254, Lithuania; ‡ Group for Molecular Engineering of Functional Material, Institute of Chemical Sciences and Engineering, École Polytechnique Fédérale de Lausanne, CH-1951 Sion, Switzerland; § Department of Physical and Inorganic Chemistry, Kaunas University of Technology, Radvilenu pl. 19, Kaunas 50254, Lithuania; ∥ Institute of Chemical Physics Vilnius University, Sauletekio al. 3, Vilnius 10257, Lithuania

## Abstract

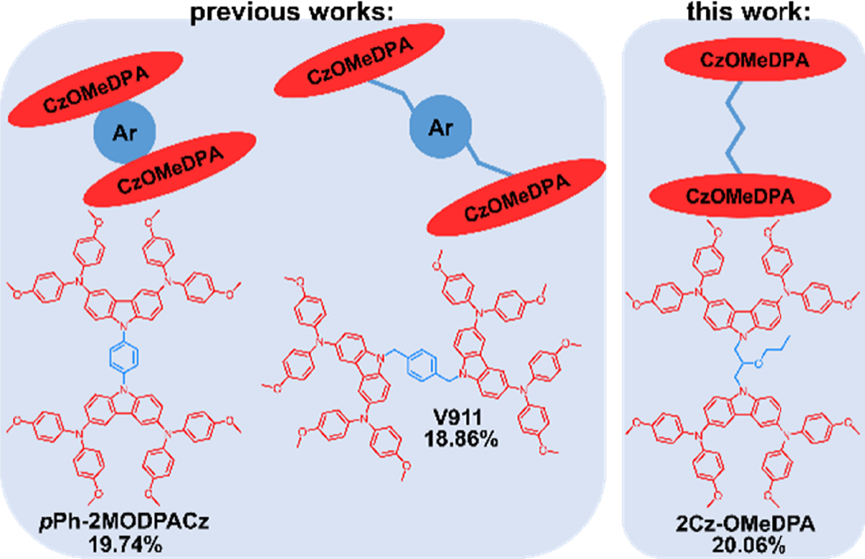

A set of novel branched molecules bearing a different number of 3,6-bis(4,4′-dimethoxydiphenylamino)carbazole-based (Cz-OMeDPA) periphery arms linked together by aliphatic chains have been developed, and their performance has been tested in perovskite solar cells (PSCs). Electrical and photovoltaic properties have been evaluated with respect to the number of Cz-OMeDPA moieties and the nature of the linking aliphatic chain. The isolated compounds possess sufficient thermal stability and are amorphous having high glass-transition temperatures (>120 °C) minimizing the risk of direct layer crystallization. The highest hole-drift mobility of μ_0_ = 3.1 × 10^–5^ cm^2^ V^–1^ s^–1^ is comparable to that of the reference standard spiro-OMeTAD (4.1 × 10^–5^ cm^2^ V^–1^ s^–1^) under identical conditions. Finally, PSCs employing two new HTMs (**2Cz-OMeDPA** and **3Cz-OMeDPA-OH**) bearing two and three substituted carbazole chromophores, linked by an aliphatic chain, show a performance of around 20%, which is on par with devices using spiro-OMeTAD and demonstrates slightly enhanced device stability.

## Introduction

Over the recent years, organic–inorganic hybrid perovskite solar cells (PSCs) have been receiving marked worldwide attention owing to their low cost and facile fabrication.^1^ Since 2009, when Miyasaka and coworkers reported a 3.8% power conversion efficiency (PCE) of PSCs,^2^ the performance of these photovoltaic devices has increased dramatically and currently, PCE exceeds 25%.^3^

A typical conventional PSC consists of a lead-halide perovskite layer sandwiched by an electron-selective layer and an organic hole-selective material, which is an important counterpart to produce high efficiency due to effective hole extraction/collection and electron blocking from the perovskite to the metal anode.^4,5^ The well-known spirobifluorene derivative 2,2′,7,7′-tetrakis-(*N*,*N*-di-*p*-methoxyphenylamine)-9,9′-spirobifluorene (spiro-OMeTAD) is the most widely used hole-transporting material (HTM) in PSCs. As spiro-OMeTAD is relatively expensive,^6^ the synthesis of novel low-cost and highly efficient HTMs is still a determinant challenge for future large-scale PSC production. Recently, HTMs representing various classes of organic compounds have been synthesized, as reviewed in numerous review articles.^7−16^

The low-cost 9*H*-carbazole as a starting material is interesting due to its excellent charge-transport properties and simple functionalization of the structure with a variety of different groups, which enable fine-tuning of the optical and electronic properties of target HTMs.^17,18^ Therefore, carbazole-based derivatives have been employed in organic light-emitting diodes^19,20^ and dye-sensitized solar cells.^21−23^ In recent years, carbazole has also attracted much attention in PSCs.^24−29^

**Figure 1:**
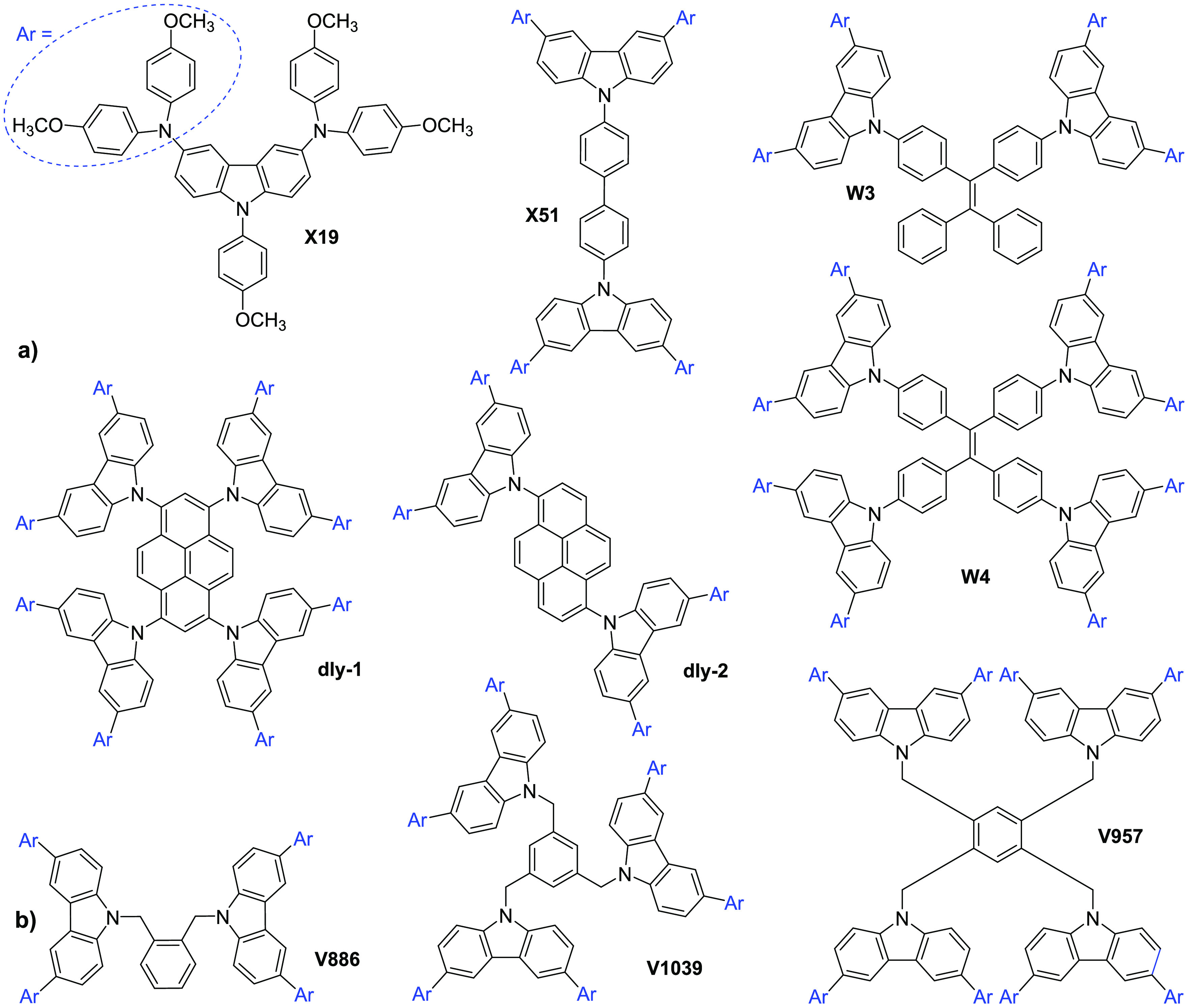
Chemical structures of the reported HTMs containing Cz-OMeDPA arms: (a) *N*-aryl substituted carbazole molecules and (b) partially nonconjugated carbazole derivatives.

In this context, the 3,6-bis(4,4′-dimethoxydiphenylamino)carbazole moiety, whose facile synthesis requires just a few steps from commercially available and cheap starting reagents, has been widely explored.^30−32^ The majority of these studies consistently demonstrate that the number of carbazole-based periphery arms in *N*-aryl substituted carbazole molecules is of crucial importance for solar cell PCE.^40^ For example, hole mobility and conductivity of X51 are higher than those of X19, leading to better photovoltaic performance of the investigated devices^30^ (Figure 1a). The results are congruous with the expected influence of the larger conjugated system in the HTM X51. However, the PCE of PSCs, in which the tetraphenylethylene-based structure with four carbazole-based periphery arms (dly-1) is employed as HTM, is lower than that of the devices with the semiconductor bearing two carbazole-based periphery arms as HTM (dly-2).^31^ In their study of *N*-aryl substituted carbazole derivatives, Wu and coworkers have demonstrated that the number of carbazole-based periphery arms significantly influences the physical properties of HTM and its application in PSCs. HTM bearing four arms (W4) has been shown to perform very well in PSCs, whereas HTM bearing just two arms (W3) was not even tested due to insufficient solubility^32^ (Figure 1a). Interestingly, a twin molecule, V886, bearing a partially nonconjugated 1,2-bismethylbenzene core has demonstrated almost state-of-the-art performance.^33^ In the consistent study of this type of compound, we have demonstrated that performance of such twin molecules can be improved by modifying carbazole-based periphery arms in the central benzene core. However, an increased number of branches in V1039 and V957 did not improve the performance^34^ (Figure 1b).

To the best of our knowledge, 3,6-bis(4,4′-dimethoxydiphenylamino)carbazole derivatives bearing photoconductive chromophores linked by aliphatic chains have not been employed in PSCs as HTMs. However, Benhattab et al. synthesized carbazole-based twin molecules linked by nonconjugated linear alkyl chains of different lengths and investigated the properties of these twin molecules in the solid-state DSSCs for the first time. They have demonstrated that conjugated linkers are not essential for designing twin molecules.^35^

Alkyl chains are attracting attention as they usually improve solubility and, therefore, enhance the pore filling of the perovskite layer by HTM forming a strong and close attachment to the perovskite to enhance charge transfer. The HTM therefore is needed to have a low tendency to crystallize, easily forming a smooth layer at the interface to favor the charge transfer. In a majority of cases, the alkyl chains are the ones to significantly influence the stability of the amorphous state, which is of crucial importance in the formation of good-quality layers.^36^

**Figure 2:**
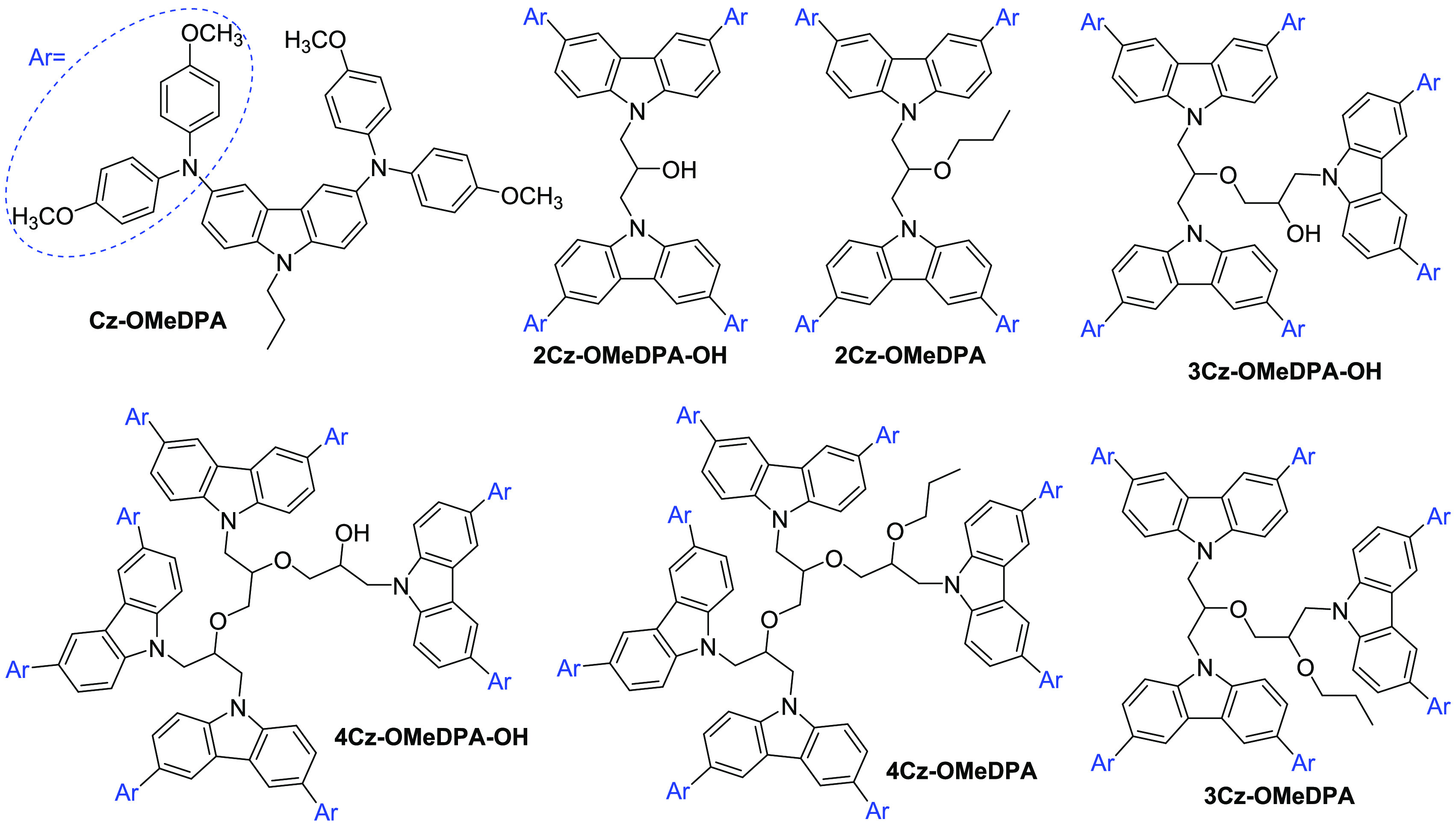
Chemical structures of the *N*-alkyl substituted carbazole HTMs containing OMeDPA arms reported herein.

Considering the above-mentioned information, we report the synthesis of branched molecules bearing a different number of 3,6-bis(4,4′-dimethoxydiphenylamino)carbazole-based (Cz-OMeDPA) periphery arms linked together by aliphatic chains (Figure 2) and investigation of their structure–property relationship. The photoelectrical and photovoltaic properties of the novel compounds in PSCs have been investigated with respect to the number of Cz-OMeDPA moieties and the nature of the linking aliphatic chain.

## Results and Discussion

### Synthesis

**Scheme 1:**
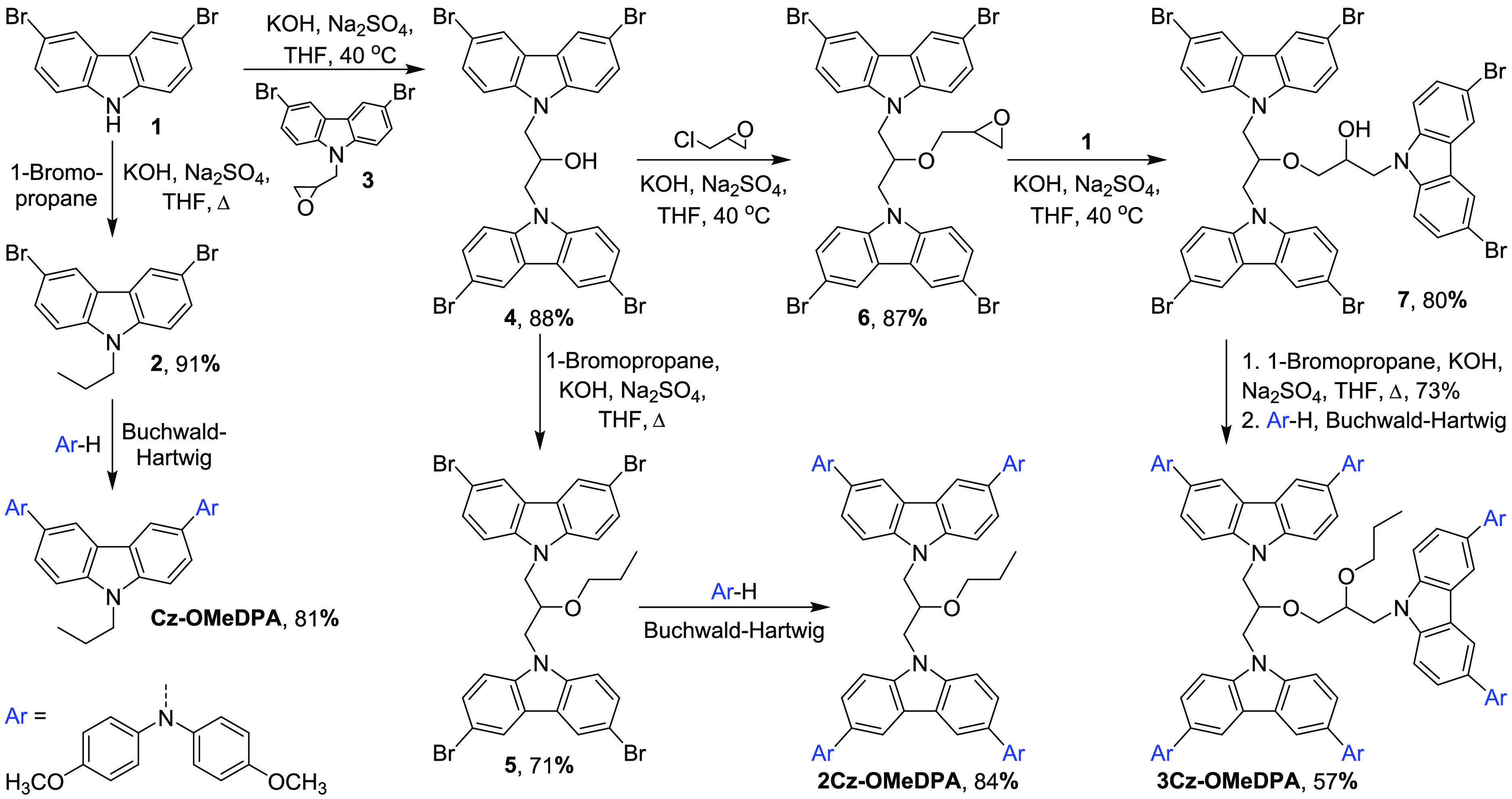
Divergent Synthesis Route to **Cz-OMeDPA**, **2Cz-OMeDPA**, and **3Cz-OMeDPA**

The target compounds bearing one (**Cz-OMeDPA)**, two (**2Cz-OMeDPA)**, or three (**3Cz-OMeDPA**) substituted carbazole chromophores linked by an aliphatic chain were synthesized according to a divergent synthesis pathway as depicted in Scheme 1. The alkylation reaction of 3,6-dibromocarbazole (**1**) with 1-bromopropane in THF in the presence of anhydrous Na_2_SO_4_ and KOH at a reflux temperature of the reaction mixture afforded 3,6-dibromo-9-propyl-9*H*-carbazole (**2**). The Buchwald–Hartwig cross-coupling reaction of intermediate **2** with bis(4-methoxyphenyl)amine provided the model compound **Cz-OMeDPA** with an extended π-electron conjugated system. With the aim of increasing the number of π-electron conjugated system by increasing the number of chromophores in the molecule, the target compound **2Cz-OMeDPA** containing two linked carbazolyl moieties was synthesized. The reaction of 3,6-dibromo-9-epoxypropylcarbazole (**3**) with 3,6-dibromocarbazole in the presence of anhydrous Na_2_SO_4_ and KOH afforded 1,3-bis(3,6-dibromo-9*H*-carbazol-9-yl)-2-propanol (**4**). However, the attempts to carry out the Buchwald–Hartwig reaction of **4** with bis(4-methoxyphenyl)amine failed. It has been assumed that the hydroxyl group in the molecule passivates the catalyst. Therefore, the hydroxyl group was replaced by the propoxy group during the alkylation reaction of **4** with 1-bromopropane to give 1,3-bis(3,6-dibromo-9*H*-carbazol-9-yl)-2-propoxypropane (**5**). The Buchwald–Hartwig reaction of intermediate **5** with bis(4-methoxyphenyl)amine afforded the target twin molecule **2Cz-OMeDPA**.

As the next step toward an increased number of photoconductive moieties, the derivative **3Cz-OMeDPA** bearing three carbazolyl chromophore molecules was synthesized. First, dimer **4** was transformed into its glycidyl ether **6**(37) via the reaction of **4** with epichlorohydrin to afford 1,3-bis(3,6-dibromo-9*H*-carbazol-9-yl)-2-(2,3-epoxy)propoxypropane (**6**). Then, the nucleophilic oxirane ring opening reaction of **6** with 3,6-dibromocarbazole in THF in the presence of anhydrous Na_2_SO_4_ and KOH provided trimer **7**. Since the Buchwald–Hartwig reaction does not occur in the presence of the hydroxyl group in the molecule, the alkylation reaction of **7** with 1-bromopropane was carried out to replace the hydroxyl group with the propoxy one in the intermediate compound, which was subsequently treated with bis(4-methoxyphenyl)amine under the conditions of the Buchwald–Hartwig reaction to obtain the target compound **3Cz-OMeDPA**.

**Scheme 2:**
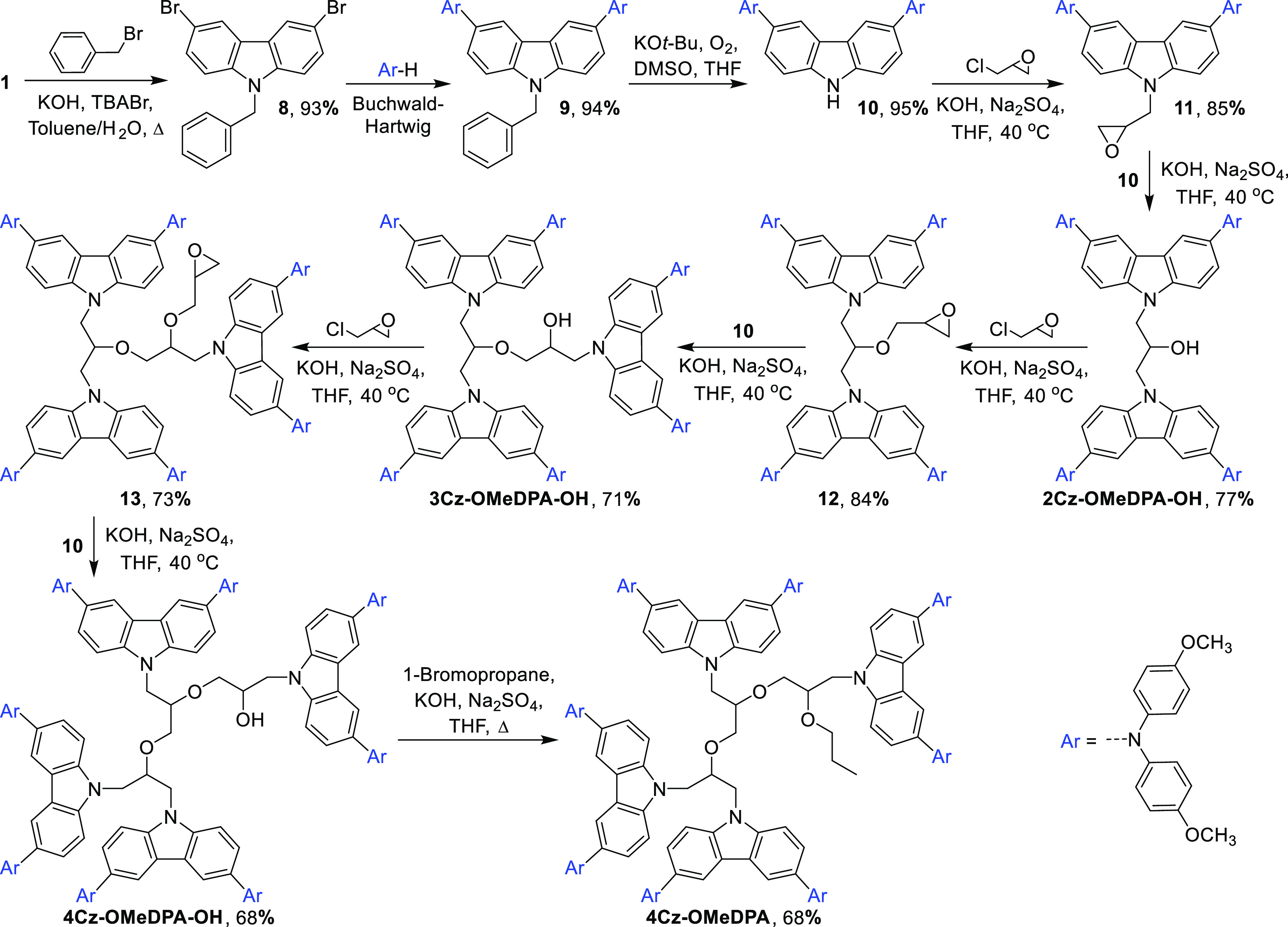
Convergent Synthesis Route to **2Cz-OMeDPA-OH**, **3Cz-OMeDPA-OH**, **4Cz-OMeDPA**, and **4Cz-OMeDPA-OH**

The attempts to synthesize a derivative bearing four substituted carbazole chromophores linked by an aliphatic chain via the divergent synthesis pathway did not yield the target compound. Therefore, this HTM was synthesized by the convergent synthesis route (Scheme 2). First, the intermediate compound 9-benzyl-3,6-dibromo-9*H*-carbazole (**8**) with the blocked N–H group was synthesized in the reaction of 3,6-dibromocarbazole (**1**) with benzyl bromide at reflux temperature of the toluene–water reaction mixture in the presence of KOH and tetrabutylammonium bromide as interphase catalyst. Next, the Buchwald–Hartwig reaction of **8** with bis(4-methoxyphenyl)amine provided 9-benzyl-3,6-bis(4,4′-dimethoxydiphenylamino)-9*H*-carbazole (**9**), which was dissolved in dimethyl sulfoxide and treated with a 1 M solution of potassium *tert*-butoxide in THF with the atmospheric oxygen participating in the reaction to give the unblocked 3,6-bis(4,4′-dimethoxydiphenylamino)-9*H*-carbazole (**10**). Compound **10** was converted into its oxirane derivative **11** in the reaction with epichlorohydrin. The next synthesis steps provided dimer **2Cz-OMeDPA-OH** bearing the OH group in the linking aliphatic chain and its glycidyl ether **12** according to the synthesis procedure depicted for compound **6** in Scheme 1. Afterward, the reaction of glycidyl ether **12** with the precursor **10** afforded trimer **3Cz-OMeDPA-OH** bearing the OH group in the aliphatic linker, which was not synthesized via a divergent synthesis route (Scheme 1). Treatment of the former compound with epichlorohydrin provided oxirane derivative **13**, which upon subsequent reaction with precursor **10** afforded the target tetramer **4Cz-OMeDPA-OH**, in which four substituted carbazole chromophores are linked by the aliphatic moiety bearing the OH group. The target compound **4Cz-OMeDPA** was synthesized by alkylating the hydroxyl group in **4Cz-OMeDPA-OH** with 1-bromopropane.

All HTMs bearing photoconductive chromophores in their molecules were purified by column chromatography followed by precipitation. Isolated by such a procedure, target products **Cz-OMeDPA**, **2Cz-OMeDPA**, **2Cz-OMeDPA-OH**, **3Cz-OMeDPA**, **3Cz-OMeDPA-OH**, **4Cz-OMeDPA**, and **4Cz-OMeDPA-OH** are amorphous compounds and all attempts to crystallize them failed. It can be assumed that the obtained high morphological stability of the synthesized compounds may be explained by the flexibility of the branched aliphatic binding chains between photoconductive chromophores. In addition, the existence of several diasteroisomers of **3Cz-OMeDPA**, **3Cz-OMeDPA-OH**, **4Cz-OMeDPA**, and **4Cz-OMeDPA-OH**, which have several chiral carbon atoms, is attributed to the stability of the amorphous state of these HTMs. The synthesized HTMs containing a different number of Cz-OMeDPA arms have a well-defined structure, and they show good room-temperature solubility in a variety of organic solvents, i.e., acetone, toluene, chlorobenzene, MEK, and THF. The structures of the HTMs have been confirmed by ^1^H NMR, ^13^C NMR, IR spectroscopy, mass spectrometry, and elemental analysis.

### Thermal Properties

**Figure 3:**
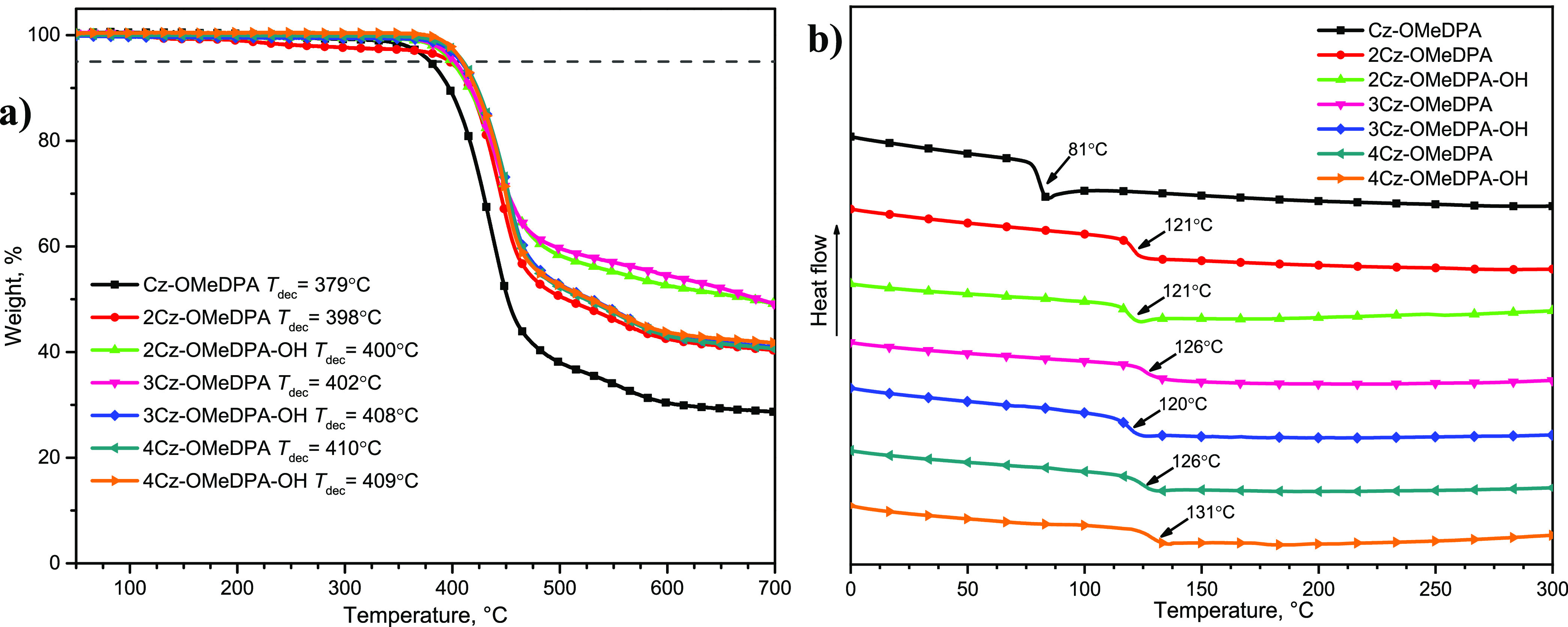
(a) TGA heating curves of the HTMs (heating rate of 10 °C/min and N_2_ atmosphere) and (b) DSC curves of the second run (heating rate of 10 °C/min and N_2_ atmosphere).

The thermal behavior of the HTMs was evaluated by thermogravimetric analysis (TGA) and differential scanning calorimetry (DSC) measurements. The data are provided in Figure 3, and the summarized characteristics are listed in Table 1. As seen from the TGA results, all compounds are thermally stable up to ∼400 °C, which is somewhat a lower temperature than that of spiro-OMeTAD (*T*_dec_ = 449 °C).^38^ The lowest 5% weight loss temperature (*T*_dec_) of 379 °C has been recorded for **Cz-OMeDPA** bearing one substituted carbazole fragment; however, it is still high enough, indicating sufficient thermal stability needed in PSCs. The highest *T*_dec_ of 410 °C has been determined for **4Cz-OMeDPA** bearing four substituted carbazole chromophores linked by the branched aliphatic chain. Overall, just small variations in *T*_dec_ values have been observed in the set of the synthesized compounds with different numbers of Cz-OMeDPA arms. However, a slight increase in *T*_dec_ values can be noticed with the increasing number of photoconductive Cz-OMeDPA arms in the synthesized HTMs. This tendency can, presumably, be explained by the higher molecular mass resulting in stronger intermolecular interactions. The same pattern has been observed for the compounds bearing the hydroxyl group in the branched aliphatic chain. Their *T*_dec_ values are slightly higher than those of the respective HTMs without hydroxyl groups. These results indicate the influence of the intermolecular hydrogen bonds.

**Table 1:** Thermal, Optical, and Photophysical Properties of the HTMs

ID	*T*_g_ (°C)^a^	*T*_dec_ (°C)^a^	λ_abs_ (nm)^b^	λ_PL_ (nm)^b^	Φ_PL_ (%)	*I*_P_ (eV)^c^	*E*_g_ (eV)^d^	*E*_ea_ (eV)^e^	μ_0_ (cm^2^ V^–1^ s^–1^)^f^
**Cz-OMeDPA**	81	379	305, 372	448	23	4.99	2.92	2.07	2.2 × 10^–9^
**2Cz-OMeDPA**	121	398	303, 372	449	18	5.16	2.97	2.19	3.2 × 10^–6^
**3Cz-OMeDPA**	126	402	303, 372	449	18	5.08	2.92	2.16	2.4 × 10^–6^
**4Cz-OMeDPA**	126	410	302, 373	452	22	5.17	2.88	2.29	1.2 × 10^–6^
**2Cz-OMeDPA-OH**	121	400	303, 372	449	18	5.12	2.94	2.18	3.1 × 10^–5^
**3Cz-OMeDPA-OH**	120	408	303, 373	450	17	5.16	2.91	2.25	6.9 × 10^–6^
**4Cz-OMeDPA-OH**	131	409	303, 373	452	19	5.18	2.82	2.36	4.0 × 10^–6^

a Glass-transition (*T*_g_) and decomposition (*T*_dec_) temperatures determined from DSC and TGA, respectively (10 °C/min and N_2_ atmosphere).

b UV–vis and PL spectra were measured in THF solutions (10^–4^ M).

c Ionization energies of the films measured using PESA.

d *E*_g_ estimated from the intersection of absorption and emission spectra of solid films.

e *E*_ea_ = *I*_P_ – *E*_g_.

f Mobility value at zero field strength.

DSC analysis has shown that all investigated compounds exist only in an amorphous state since no endothermic melting peaks were detected during both heating cycles (Figures S1–S7). From the data in Figure 3b and Table 1, it can be found that the molecules bearing a different number of Cz-OMeDPA moieties show just insignificant variations in *T*_g_, i.e., 121–131 °C. The only notable exception is *T*_g_ of the compound **Cz-OMeDPA** bearing just one substituted carbazole chromophore. Its *T*_g_ is much lower (*T*_g_ = 81 °C) than those of the compounds bearing a greater number of carbazole fragments. With an increase in the number of substituted carbazole chromophores, the *T*_g_ values for **3Cz-OMeDPA**, **4Cz-OMeDPA**, and **4Cz-OMeDPA-OH** are also increased, leading to a stabilized amorphous state compared with spiro-OMeTAD (124 °C). In general, it is advantageous to use fully amorphous compounds as there is no risk of direct film crystallization in photovoltaic devices.^38^

### Optical Properties

**Figure 4:**
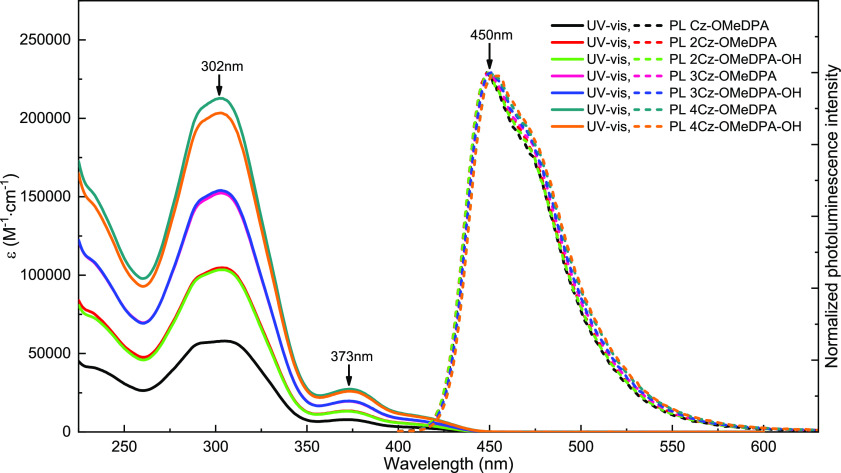
UV–vis absorption (solid line) and photoluminescence (dashed line) spectra of the investigated HTMs in THF solution (10^–4^ M).

The optical properties were evaluated by UV–vis absorption and photoluminescence spectroscopy in dilute THF solutions and thin films on a glass substrate. UV–vis spectra of the synthesized compounds in solution are similar, as strong π–π* absorptivity is observed at 270–350 nm, and weaker energy absorption, which can be assigned to *n*–π* bands, is present at ∼373 nm (Figure 4). The different number of carbazole fragments in the molecules does not influence conjugation; just the hyperchromic effect is noted in the series starting from **Cz-OMeDPA**, through **2Cz-OMeDPA** and **3Cz-OMeDPA**, to **4Cz-OMeDPA**. A similar trend was observed for the compounds **2Cz-OMeDPA-OH**, **3Cz-OMeDPA-OH**, and **4Cz-OMeDPA-OH**. Thus, absorptivity is directly proportional to the number of Cz-OMeDPA moieties in the synthesized compounds. This relationship has been definitely proven once again by the structures of the synthesized HTM molecules. Changes in the branched aliphatic chains linking photoconductive chromophores do not influence the absorption spectra of the target compounds. The same pattern applies to the characteristics of the materials investigated in the thin films. Significant changes in the UV–vis spectra have not been observed in the case of the films on the glass substrate (Figure S8). All investigated HTMs emitted light with a maximum at around 450 nm and a photoluminescence quantum yield (Φ**_PL_**) of ∼20% in THF solutions estimated using the integrated sphere method.^41^ In addition, a relatively large Stokes shift of ∼80 nm was observed for the synthesized compounds, which suggests significant changes in the geometry of the photoconductors upon excitation. We next calculated the optical gaps (*E*_g_ = 2.9 eV) from the intersection of UV–vis and PL spectra on glass substrates.

### Photoelectrical Properties

**Figure 5:**
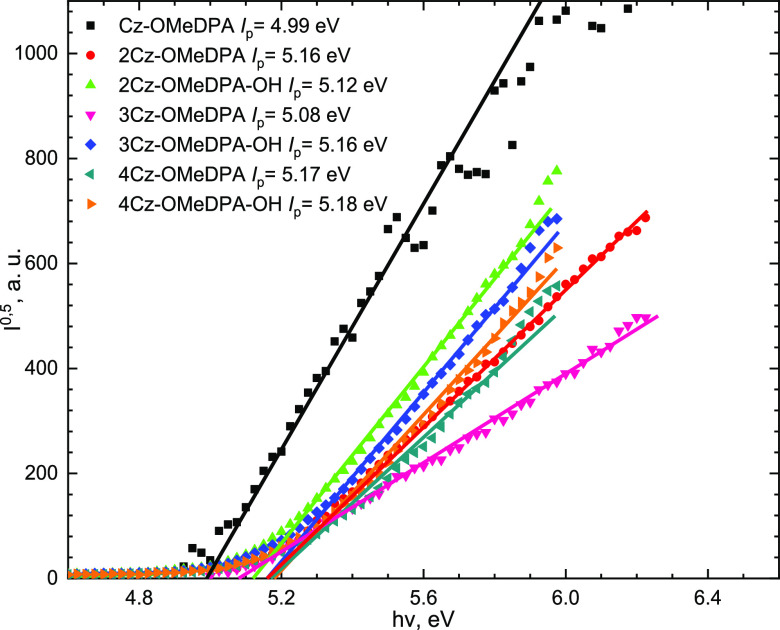
Photoemission in air spectra of HTMs.

To better understand the HOMO–LUMO level alignment of the synthesized HTMs in PSCs, the solid-state ionization potential (*I*_p_) of their thin films was recorded by photoelectron emission spectroscopy in air (PESA), as shown in Figure 5. As it has been expected, among the molecules bearing the same chromophores linked together by aliphatic chains, only insignificant variations were detected, with the lowest value of 4.99 eV measured for **Cz-OMeDPA** and the highest one of 5.18 eV recorded for **4Cz-OMeDPA-OH**. In all cases, the *I*_p_ values are similar to that of spiro-OMeTAD (5.00 eV), which optimally offsets with the perovskite valence band energy (∼5.5 eV); therefore, effective hole transport from the photoactive perovskite to the electrode should be fulfilled.

**Figure 6:**
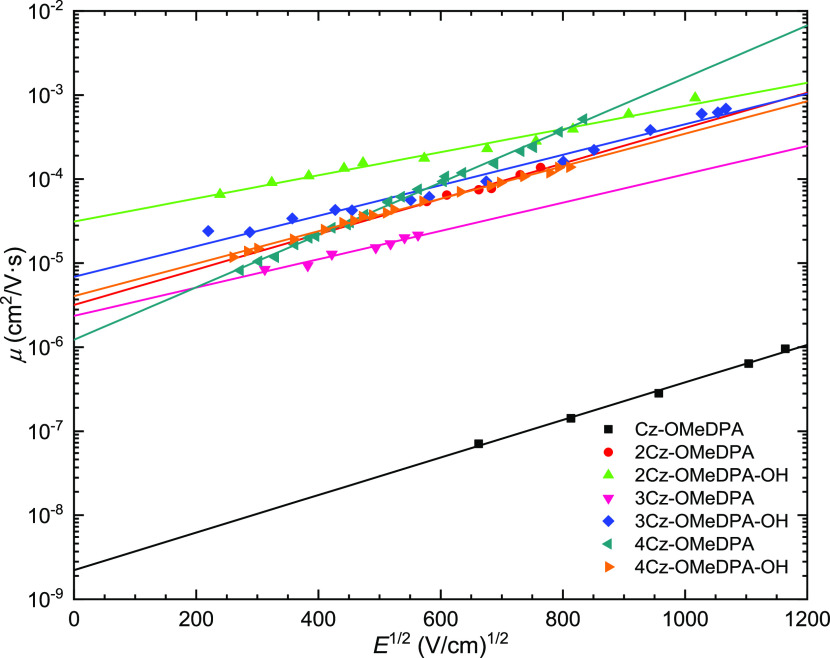
Electric field dependencies of the hole-drift mobility (μ) in charge transport layers of investigated HTMs.

Furthermore, the charge mobility of the synthesized HTMs was recorded using the xerographic time of flight (XTOF) technique for solution-processed films (for more details, see the Methods section) measuring hole-drift mobility on electric field strength dependency. The results obtained are shown in Figure 6. As shown in Table 1, in the series of the branched derivatives, the zero-field hole-drift mobility (μ_0_) of 3.1 × 10^–5^ cm^2^ V^–1^ s^–1^ for **2Cz-OMeDPA-OH** is the highest, while that of the analogue with alkylated hydroxyl group **2Cz-OMeDPA** is an order of magnitude lower (3.2 × 10^–6^ cm^2^ V^–1^ s^–1^). Interestingly, a larger number of photoconductive chromophores have a negative influence on the hole-drift mobility of the synthesized HTMs. The hole-drift mobility of **3Cz-OMeDPA-OH** with three Cz-OMeDPA moieties is somewhat lower than that of **2Cz-OMeDPA-OH**, while the value for HTM with four Cz-OMeDPA moieties (**4Cz-OMeDPA-OH**) was even lower at weak electric fields. The same pattern has been noticed among the HTMs with alkylated hydroxyl groups **2Cz-OMeDPA**, **3Cz-OMeDPA**, and **4Cz-OMeDPA**. It is important to note that the hole-drift mobility value of **Cz-OMeDPA** is much lower (2.2 × 10^–9^ cm^2^ V^–1^ s^–1^), showing that, in the present case, the nonbranched molecular structure is disadvantageous for the efficient hole transport.

The observed drop in charge mobility may be explained by the less ordered packing of the more branched molecules with a larger number of side groups since charges are hopping between molecules spaced further apart. Usually, the mobility in the molecular solids is calculated according to the Borsenberger, Pautmeier, and Bässler formula^39^
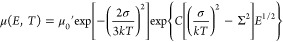
1

Here, μ is the hole-drift mobility; μ_0_′ is the mobility prefactor; σ is the energy width of the hopping site manifold, which is a measure of the energetic disorder; Σ is the degree of positional disorder; *C* is the empirical constant of 2.9 × 10^–4^ (cm V^–1^)^0.5^; *E* is the electric field; and *kT* has its usual meaning. As seen from formula 1, the zero-electric field mobility μ(0, *T*) is determined mainly by the energetic disorder σ; therefore, we may assume that energetic disorder is lower in the case of less-branched HTMs bearing two or three Cz-OMeDPA moieties as compared to the analogous compounds with four branches having the highest steric disorder. Finally, the hole mobility of **2Cz-OMeDPA-OH** is equivalent to that of the reference standard spiro-OMeTAD (4.1 × 10^–5^ cm^2^ V^–1^ s^–1^).^38^

### Photovoltaic Properties

**Figure 7:**
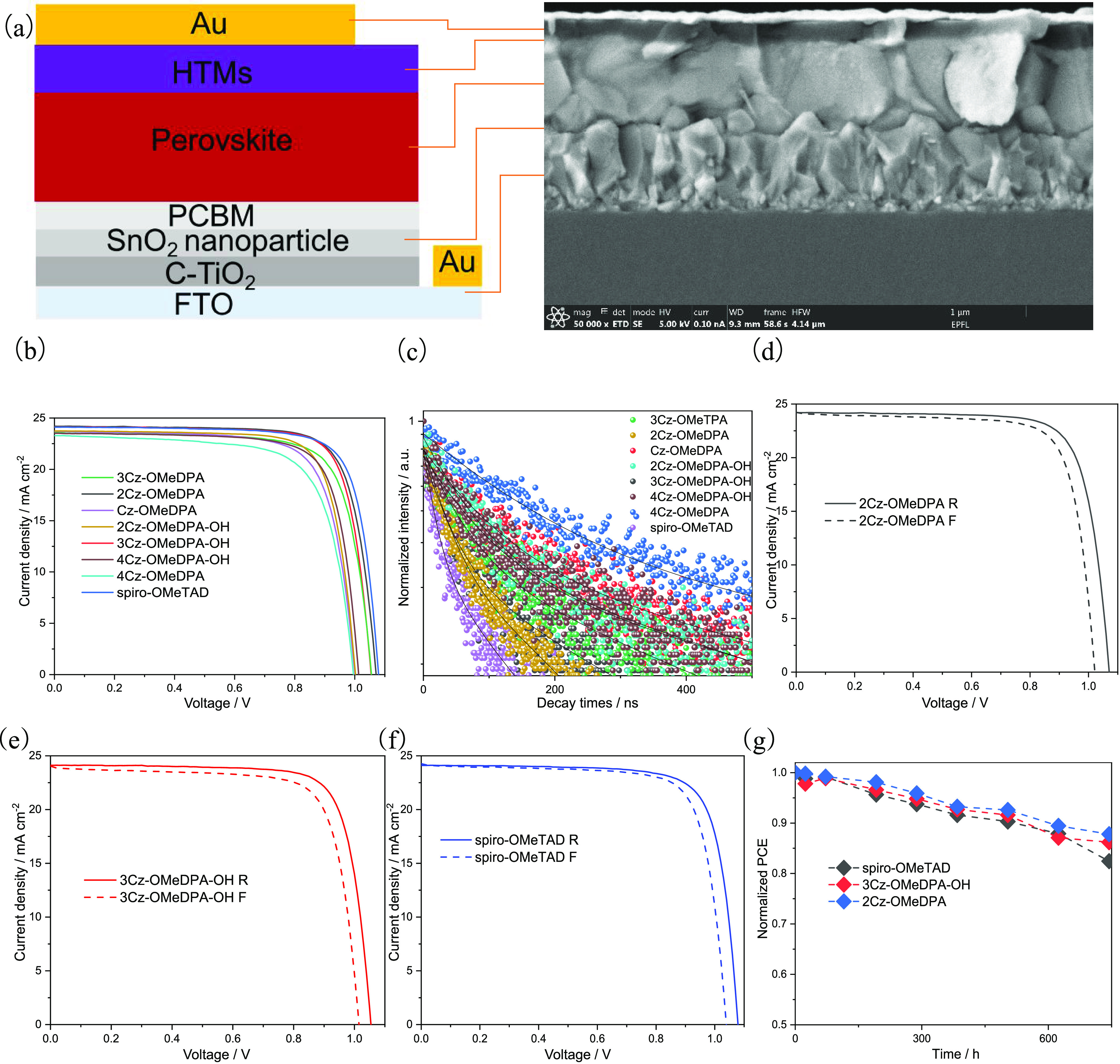
(a) Illustration of the devices constructed from FTO/C-TiO_2_/SnO_2_/PCBM/perovskite/HTM/Au, along with the corresponding cross-sectional SEM image; (b) champion *J*–*V* curves of various HTMs based on PSC; (c) TRPL of various HTMs in the construction of glass/perovskite/HTM; reverse and forward scan of *J*–*V* curves based on (d) **2Cz-OMeDPA**, (e) **3Cz-OMeDPA-OH**, and (f) spiro-OMeTAD; and (g) stability of devices based on spiro-OMeTAD, **3Cz-OMeDPA-OH**, and **2Cz-OMeDPA** (the humidity is lower than 10%, N_2_-filled box).

The n–i–p PSCs with the architecture FTO/C-TiO_2_/SnO_2_/PCBM/perovskite/HTM/Au were fabricated, as shown in Figure 7a (the details are given in the Methods section). The FAMAPbI_3_ dominated the perovskite composition, and the HTM layers were doped. The thickness of the corresponding layers was determined by cross-sectional scanning electron microscopy (SEM). The device was made by layering 700 nm perovskite atop a thin SnO_2_ nanoparticle layer, which was deposited on FTO glass coated with compact TiO_2_. The device was completed by 70 nm thick HTL and 70 nm gold as back contacts.

The current–voltage (*J*–*V*) traces of the record devices of each HTM are provided in Figure 7b, and their PV parameters are summarized in Table 2; also, the statistics of PCE are presented in Figure S11. The PCE values of the most efficient devices containing **2Cz-OMeDPA**, **3Cz-OMeDPA-OH**, and spiro-OMeTAD were very similar, i.e., 20.06%, 19.89%, and 20.25%, respectively. The hysteresis of the devices was evaluated based on the *J*–*V* curves collected by scanning the device from forward bias (FB) to the short circuit (SC) named as F followed by scanning from SC to FB named as R (Figures 7d–f and S9). The data has revealed that the devices based on new HTMs of **2Cz-OMeDPA** and **3Cz-OMeDPA-OH** exhibited similar hysteretic behavior as compared to spiro-OMeTAD.

**Table 2:** Photovoltaic Parameters of Reverse (R) and Forward (F) Scans Obtained from the Champion Devices Based on Various HTMs

HTMs	*J*_sc_ (mA cm^–2^)	*V*_oc_ (V)	FF (%)	PCE (%)
**Cz-OMeDPA R**	23.62	1.009	74.3	17.77
**Cz-OMeDPA F**	23.35	0.985	72.6	16.73
**2Cz-OMeDPA R**	24.15	1.071	77.4	20.06
**2Cz-OMeDPA F**	24.16	1.021	76.6	18.95
**2Cz-OMeDPA-OH R**	23.7	1.001	77.2	18.33
**2Cz-OMeDPA-OH F**	23.24	0.971	73.4	16.60
**3Cz-OMeDPA R**	23.59	1.054	75.1	18.72
**3Cz-OMeDPA F**	23.26	1.004	74.2	17.38
**3Cz-OMeDPA-OH R**	24.05	1.054	78.3	19.89
**3Cz-OMeDPA-OH F**	24.07	1.015	75.9	18.53
**4Cz-OMeDPA-OH R**	23.51	1.012	76.3	18.15
**4Cz-OMeDPA-OH F**	23.61	0.988	76	17.78
**4Cz-OMeDPA R**	23.36	0.993	71.1	16.55
**4Cz-OMeDPA F**	22.16	0.946	67.5	14.19
spiro-OMeTAD R	24.05	1.079	77.9	20.25
spiro-OMeTAD F	24.22	1.039	76.7	19.33

To realize the performance difference between the HTMs, time-resolved photoluminescence (TRPL) measurements (Figure 7c) based on glass/perovskite/HTMs construction were performed to study the decay to hole injection processes, as well as the decay time was fitted by the bi-exponential model with the fast (τ_1_) and slow (τ_2_) components, which indicated the interfacial transportation and recombination; the average delay time (τ_ave_) is calculated by , where *A*_i_ and τ_i_ represent the decay amplitude and components of delay time, respectively. For the interfacial transportation, the fast (τ_1_) components were considered. As expected, a significant quenching is visible in the first 50 ns for the most efficient HTMs. The derived time constants of 21.3 and 43.6 ns were retrieved for **2Cz-OMeDPA** and **3Cz-OMeDPA-OH**, respectively, and a somewhat faster process with τ = 16.8 ns was obtained for the spiro-OMeTAD/perovskite interface (Table S1). On the contrary, a long-living component was observed for the least efficient compounds. These results have indicated that all of the HTMs can assist the hole transportation and **2Cz-OMeDPA** is the best among HTMs for the hole collection, which is just a little less for the hole transportation efficiency than spiro-OMeTAD. The results may be the reason for the slightly lower PCE of **2Cz-OMeDPA** compared with that of spiro-OMeTAD.

As shown in Figure 7g, the stability of the unencapsulated devices containing the best performing HTMs was measured under 1 sun illumination while stored under a N_2_ atmosphere. Devices containing **2Cz-OMeDPA** are the most stable, and their stability was slightly better than that of the devices containing **3Cz-OMeDPA-OH** and spiro-OMeTAD.

## Conclusions

To conclude, the synthesis and a systematic study of the branched molecules bearing a different number of 3,6-bis(4,4′-dimethoxydiphenylamino)carbazole (Cz-OMeDPA) in the periphery linked by aliphatic chains as hole-transporting materials for PSCs are reported. The influence of the different number of Cz-OMeDPA fragments has been revealed through the optical, electrochemical, photophysical, and photovoltaic measurements. Notably, the molecular engineering of Cz-OMeDPA arms resulted in the charge drift mobility of μ_0_ = 3.1 × 10^–5^ cm^2^ V^–1^ s^–1^, which is comparable to that of the reference standard spiro-OMeTAD (4.1 × 10^–5^ cm^2^ V^–1^ s^–1^) under identical conditions. Most importantly, PSCs employing **2Cz-OMeDPA** bearing two carbazole chromophores showed a performance of over 20%, which is the best result among the series being on par with spiro-OMeTAD, and demonstrated the enhanced device stability.

## Methods

### Ionization Potential Measurements

The solid-state ionization potential (*I*_p_) was measured according to the electron photoemission in air^42−44^ by dissolving HTMs in THF and coating layers of 0.5–1 μm thickness on the Al plate, which was precoated with methyl methacrylate and methacrylic acid copolymer adhesive layers (∼0.5 μm thick). Samples were illuminated with monochromatic light originating from a quartz monochromator with a deuterium lamp. The power of the incident light beam was 2–5 × 10^–8^ W. A negative voltage of −300 V was supplied to the sample substrate. A counter electrode with a 4.5 × 15 mm^2^ slit for illumination was placed at a distance of 8 mm from the sample surface. For the photocurrent measurement, the counter electrode was connected to the input of the BK2-16 type electrometer working in the open input regime. The strength of the photocurrent in the circuit under illumination was 10^–15^–10^–12^ A. The photocurrent *I* depends on the incident light photon energy *h*ν. The *I*^0.5^ = *f*(*h*ν) dependence was plotted. The dependence of the photocurrent on incident light quanta energy is described by a linear relationship between *I*^0.5^ and *h*ν near the threshold. The linear part of this dependence was extrapolated to the *h*ν axis, and the *I*_p_ value was determined as the photon energy at the interception point.

### Hole-Drift Mobility Measurements

Samples were prepared by spin-coating the HTM solution on the polyester film with a conductive Al layer. The thickness of the spin-coated layer was 5–10 μm. The hole-drift mobility was measured by XTOF.^45,46^ The electric field was created by positive corona charging. Charge carriers were generated at the layer surface by illumination with pulses of the nitrogen laser (pulse duration, 2 ns; wavelength, 337 nm). The layer surface potential decreased up to 1–5% of initial potential before illumination as a result of pulse illumination. The capacitance probe connected to the wide frequency band electrometer measured the speed of the surface potential decrease d*U*/d*t*. The transit time *t*_t_ was determined by the kink on the curve of the d*U*/d*t* transient on a double logarithmic scale. Drift mobility was calculated according to the formula μ = *d*^2^/*U*_0_*t*_t_, where *d* is the layer thickness and *U*_0_ is the surface potential at the moment of illumination.

### Thermal Properties

DSC was performed with a Q10 calorimeter (TA Instruments) at a scan rate of 10 K min^–1^ under a nitrogen atmosphere. The glass-transition temperature of each synthesized compound was determined during the second heating scan. TGA was performed with a Q50 TGA (TA Instruments) at a scan rate of 10 K min^–1^ under a nitrogen atmosphere.

### Device Fabrication

The chemically etched FTO glass (Nippon Sheet Glass) was cleaned with detergent solution, followed by acetone and then ethanol. The C-TiO_2_ layer was prepared by spraying TAA solution in ethanol (0.2 mL of TAA in 6 mL of anhydrous ethanol) at 450 °C. SnO_2_ nanoparticles were diluted with deionized water in a ratio of 1:4 and coated on the C-TiO_2_ substrate at a speed of 3000 rpm for 20 s with a ramp-up of 2000 rpm s^–1^ followed by the final heating at 150 °C for 10 min. A 10 mg/mL concentration solution of PCBM in chlorobenzene was prepared and was spin-coated on the SnO_2_ layer at a speed of 3000 rpm for 20 s with a ramp-up of 2000 rpm s^–1^ followed by the final heating at 100 °C for 10 min. Afterward, perovskite solutions (the ratio of PbI_2_, MAI, FAI, and PbBr_2_ was 1:0.16:0.84:0.11, and 1.38 mmol/mL PbI_2_ solution and 0.305 mmol/mL MACl solution were added to the perovskite solution; the solvent was prepared by mixing DMSO and DMF in a ratio of 1:4) were successively spin-coated on the substrates at 1000 rpm for 10 s and 5000 rpm for 30 s, respectively. Chlorobenzene (200 μL) was added dropwise for 10 s at 5000 rpm. Perovskite films were annealed at 150 °C for 10 min. The control HTM solution was prepared by dissolving 75 mg of spiro-OMeTAD (Merck) and additives in 1 mL of chlorobenzene. For each sample solution of the synthesized HTMs, 50 mg of the compound was dissolved in 1 mL of chlorobenzene. Li-bis(trifluoromethanesulfonyl)imide (18 μL) from the stock solution (520 mg in 1 mL of acetonitrile), 13 μL of FK209 [tris (2-(1*H*-pyrazol-1-yl)-4-*tert*-butylpyridine)-cobalt(III) tris(bis (trifluoromethylsulfonyl)imide) (375 mg in 1 mL of acetonitrile)], and 30 μL of 4-*tert*-butylpyridine were added as additives. The HTM layer was formed by spin-coating the solution at 4000 rpm for 20 s. As the final step, the 70 nm thick Au electrode was deposited by thermal evaporation. All preparative work to deposit PCBM, perovskite, and HTMs was performed inside the glove box under nitrogen to minimize the influence of moisture and oxygen.

### Device Characterization

The SEM of the film morphology was investigated by using a high-resolution SEM (Merlin, Zeiss) equipped with a GEMINI II column and a Schottky Field Emission gun. Images were acquired with an in-lens secondary electron detector. For the PL lifetime measurements, samples were excited with a 408 nm pulsed laser (MDL 300, PicoQuant) with a pulse energy density of 40 μm cm^–2^. Current–voltage characteristics were recorded by applying an external potential bias to the cell while recording the generated photocurrent with a digital source meter (Keithley Model 2400). The light source was a 450 W xenon lamp (Oriel) equipped with a Schott K113 Tempax sunlight filter (Praezisions Glas & Optik GmbH) to match the emission spectrum of the lamp to the AM1.5G standard. Before each measurement, the exact light intensity was determined using a calibrated Si reference diode equipped with an infrared cutoff filter (KG-3, Schott). The cells were masked with an active area of 0.09 cm^2^ to fix the active area and reduce the influence of the scattered light for the small device. All measurements were carried out at room temperature in air.

## Supporting Information Available

General procedures, synthetic methods, additional figures, and tables (DSC, UV-PL, hysteresis, fitting parameters, etc.) (PDF)
