# Supplementary material for: Branched Methoxydiphenylamine-Substituted Carbazole Derivatives for Efficient Perovskite Solar Cells: Bigger Is Not Always Better
Source: Chem Mater. 2021 Aug 19;33(17):7017–27. doi: 10.1021/acs.chemmater.1c02114 (PMC8444345; doi:10.1021/acs.chemmater.1c02114)
Supplement: Supplementary file 1 — cm1c02114_si_001.pdf [file cm1c02114_si_001.pdf]

Supporting Information

Branched Methoxydiphenylamine-Substituted Carbazole Derivatives for Efficient Perovskite Solar Cells: Bigger Isn't Always Better

Povilas Luizys,[1] Jianxing Xia,[2] Maryte Daskeviciene,[1] Kristina Kantminiene,[3] Ernestas Kasparavicius,[1] Hiroyuki Kanda,[2] Yi Zhang,[2] Vygintas Jankauskas,[4] Kasparas Rakstys,[1] Vytautas Getautis*[1] and Mohammad Khaja Nazeeruddin*[2]

[1]Department of Organic Chemistry, Kaunas University of Technology, Radvilenu pl. 19, Kaunas 50254, Lithuania

[2]Group for Molecular Engineering of Functional Material, Institute of Chemical Sciences and Engineering, École Polytechnique Fédérale de Lausanne, CH-1951 Sion, Switzerland

[3]Department of Physical and Inorganic Chemistry, Kaunas University of Technology, Radvilenu pl. 19, Kaunas 50254, Lithuania

[4]Institute of Chemical Physics Vilnius University, Sauletekio al. 3, Vilnius 10257, Lithuania

General procedures. Chemicals required for the synthesis were purchased from Sigma-Aldrich and TCI Europe. Reaction course was monitored by thin-layer chromatography on ALUGRAM SIL G/UV254 plates visualized under UV light. Silica gel (grade 9385, 230–400 mesh, 60 Å, Aldrich) was used for column chromatography. Melting points were measured by Electrothermal MEL-TEMP capillary melting point apparatus. Elemental analysis was performed by an Exeter Analytical CE-440 elemental analyzer, Model 440 C/H/N/. ^{1}H NMR spectra were recorded on a Bruker Avance III spectrometer at 400 MHz with a 5 mm double resonance broad band BBO z-gradient room temperature probe, ^{13}C NMR spectra were recorded using the same instrument at 101 MHz. The chemical shifts (expressed in ppm) are relative to tetramethylsilane (TMS). All NMR experiments were performed at 25 °C. FT-IR spectra ($\bar{\upsilon}$, cm^{-1}) were recorded by using a Perkin–Elmer Frontier spectrophotometer with a single reflectance horizontal ATR (Attenuated Total Reflectance) cell equipped with a diamond crystal. The data were recorded in the spectral range from 650 to 4000 cm^{-1} by accumulating 5 scans with a resolution of 4 cm^{-1}. UV-vis spectra were recorded on Shimadzu UV-VIS spectrophotometer UV-2600. The photoluminescence emission spectra were recorded on Edinburgh Instruments FLS920 spectrophotometer. 4-*Tert*-butylpyridine (tbp), bis(trifluoromethane) sulfonamide lithium salt, titanium diisopropoxide bis(acetylacetonate) (TAA), tin(IV) chloride pentahydrate, FK209 [tris(2-(1*H*-pyrazol-1-yl)-4-*tert*-butylpyridine)-cobalt(III) tris(bis(trifluoromethylsulfonyl) imide)], dimethyl sulfoxide (DMSO), chlorobenzene (CB), and dimethylformamide (DMF) were supplied from Sigma-Aldrich. FAI, MAI and $PbBr_2$ were acquired from GreatCell Solar. Lead iodide was obtained from Alfa Aesa. spiro-OMeTAD was acquired from Merck. PCBM was obtained from lumintac. All purchased chemicals were used as received without further purification.

3,6-Dibromo-9-propyl-9*H*-carbazole (2)

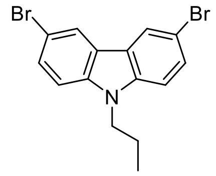

To a solution of 3,6-dibromocarbazole (0.65 g, 2 mmol) in tetrahydrofuran (32.2 mL), 1-bromopropane (5.4 mL, 60 mmol) was added. Afterwards anhydrous Na_2SO_4 (0.11 g, 0.8 mmol) and 85% powdered KOH (0.34 g, 6 mmol) were added. The reaction mixture was heated at reflux for 2.5 h. After completion of the reaction (TLC, acetone:*n*-hexane, 1:4, v:v), the reaction mixture was extracted with ethyl acetate. The organic layer was dried over anhydrous Na_2SO_4, filtered and the solvent was distilled off under reduced pressure. Ethanol was poured onto the crude residue. The formed precipitate was filtered off, dried, and used for the further synthesis without additional purification. Yield 0.67 g (90.7%).

^{1}H NMR (400 MHz, DMSO–d_6) δ, ppm: 8.46 (s, 2H); 7.64–7.55 (m, 4H); 4.34 (t, J = 7.0 Hz, 2H); 1.75 (m, 2H); 0.82 (t, J = 7.3 Hz, 3H); ^{13}C NMR (101 MHz, DMSO–d_6) δ, ppm: 139.13; 128.76; 123.39; 122.86; 111.68; 111.20; 43.92; 21.75; 11.22. Anal. calcd for $C_{15}H_{13}Br_2N$, %: C, 49.08; H, 3.57; N, 3.82. Found, %: C, 48.91; H, 3.53; N, 3.73.

3,6-Bis(4,4'-dimethoxydiphenylamine)-9-propyl-9*H*-carbazole (Cz-OMeDPA)

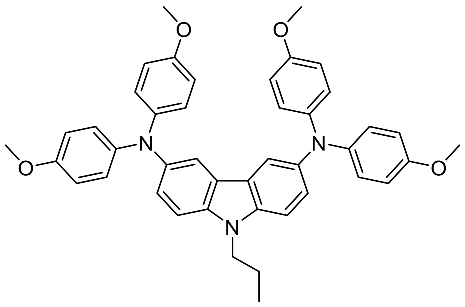

A solution of **1** (0.37 g, 1 mmol) and 4,4′-dimethoxydiphenylamine (0.69 g, 3 mmol) in dry toluene (5.3 ml) was purged with argon for 30 min. Afterwards palladium(II) acetate (0.004 g, 0.02 mmol) and tri-*tert*-butylphosphonium tetrafluoroborate (0.008 g, 0.027 mmol) were added into a mixture and it was purged with argon for 10 min. Then sodium *tert*-butoxide (0.58 g, 6 mmol) was added and the reaction mixture was heated at reflux under argon atmosphere for 17 h. After completion of the reaction (TLC, acetone:*n*-

hexane, 1:4, v:v), the reaction mixture was filtered through Celite and extracted with ethyl acetate. The organic layer was dried over anhydrous Na_2SO_4, filtered and the solvent was distilled off under reduced pressure. The crude product was purified by column chromatography (acetone:tetrahydrofuran:*n*-hexane, 1:3:46, v:v:v). Ethanol was poured onto the crude residue. The formed precipitate was filtered off and dried. Yield 0.54 g (81.0%).

^{1}H NMR (400 MHz, acetone–d_6) δ, ppm: 7.62 (br s, 2H); 7.45 (d, *J* = 8.7 Hz, 2H); 7.15 (dd, *J* = 8.7, 1.9 Hz, 2H); 6.91 (d, *J* = 8.9 Hz, 8H); 6.78 (d, *J* = 9.0 Hz, 8H); 4.32 (t, *J* = 7.1 Hz, 2H); 3.72 (s, 12H); 1.88 (m, 2H); 0.97 (t, *J* = 7.4 Hz, 3H); ^{13}C NMR (101 MHz, acetone–d_6) δ, ppm: 155.75; 143.54; 141.65; 138.51; 125.13; 125.01; 124.08; 117.38; 115.32; 110.74; 55.65; 45.17; 23.15; 11.90. FT-IR, $\bar{\upsilon}$ (cm^{-1}): 3038 (aromatic CH); 2994, 2930, 2904, 2875, 2831 (aliphatic CH); 1606, 1575, 1499, 1482, 1462, 1439 (C=C); 1233, 1215, 1033 (C–O–C). Anal. calcd for $C_{43}H_{41}N_3O_4$, %: C, 77.80; H, 6.23; N, 6.33. Found, %: C, 77.79; H, 6.38; N, 6.13. $C_{43}H_{41}N_3O_4$[M+] exact mass = 663.310, MS (MALDI-TOF) = 663.311.

1,3-Bis(3,6-dibromo-9*H*-carbazol-9-yl)-2-propanol (4)

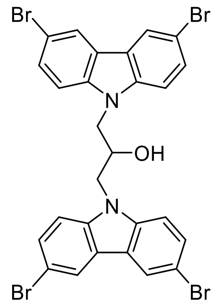

To a solution of 3,6-dibromocarbazole (4.88 g, 15 mmol) and 3,6-dibromo-9-(oxiran-2-ylmethyl)-9*H*-carbazole (**3**) (5.72 g, 15 mmol) in tetrahydrofuran (56.8 mL), anhydrous Na_2SO_4 (0.85 g, 0.6 mmol) and 85% powdered KOH (1.68 g, 30 mmol) were added. The reaction mixture was stirred at 40 °C for 18 h. After completion of the reaction (TLC, acetone:*n*-hexane, 3:22, v:v), the reaction mixture was extracted with ethyl acetate. The organic layer was dried over anhydrous Na_2SO_4, filtered and the solvent was distilled off under reduced pressure. The crude product was purified by column chromatography (acetone:*n*-hexane, 3:22, v:v). 20% solution of the residue in tetrahydrofuran was poured with intensive stirring into a 10-fold excess of ethanol. The precipitate was filtered off, washed with ethanol, and dried. Yield 9.32 g (88.0%). ^{1}H NMR (400 MHz, tetrahydrofuran–d_8) δ, ppm: 8.28 (br s, 4H,); 7.52 (dd, *J* = 8.7, 1.7 Hz, 4H); 7.45 (d, *J*

= 8.7 Hz, 4H); 4.88 (br s, 1H); 4.53–4.44 (m, 5H); ^{13}C NMR (101 MHz, tetrahydrofuran–d_8) δ, ppm: 141.18; 129.92; 124.80; 124.23; 113.10; 112.42; 70.34; 48.66. Anal. calcd for $C_{27}H_{18}Br_4N_2O$, %: C, 45.93; H, 2.57; N, 3.97. Found, %: C, 45.88; H, 2.56; N, 3.77.

1,3-Bis(3,6-dibromo-9*H*-carbazol-9-yl)-2-propoxypropane (5)

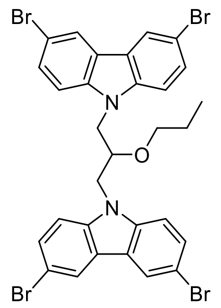

To a solution of **3** (2.12 g, 3 mmol) in tetrahydrofuran (17.5 mL), 1-bromopropane (8.1 mL, 90 mmol) was added. Afterwards anhydrous Na_2SO_4 (0.17 g, 1.2 mmol) and 85% powdered KOH (0.5 g, 9 mmol) were added and the reaction mixture was heated at reflux for 40 h. After completion of the reaction (TLC, acetone:toluene:*n*-hexane, 3:10:12, v:v:v), the reaction mixture was extracted with ethyl acetate. The organic layer was dried over anhydrous Na_2SO_4, filtered and the solvent was distilled off under reduced pressure. The crude product was purified by column chromatography (tetrahydrofuran:*n*-hexane, 3:22, v:v). 20% solution of the residue in acetone was poured with intensive stirring into a 10-fold excess of ethanol. The precipitate was filtered off, washed with ethanol, and dried. Yield 1.6 g (71.1%).

^{1}H NMR (400 MHz, tetrahydrofuran–d_8) δ, ppm: 8.29 (br s, 4H); 7.53 (dd, *J* = 8.7, 1.4 Hz, 4H); 7.43 (d, *J* = 8.7 Hz, 4H); 4.62–4.52 (m, 2H); 4.52–4.43 (m, 2H); 4.25–4.15 (m, 1H); 2.71 (t, *J* = 6.5 Hz, 2H); 0.85 (m, 2H); 0.29 (t, *J* = 7.4 Hz, 3H); ^{13}C NMR (101 MHz, tetrahydrofuran–d_8) δ, ppm: 140.95; 130.01; 124.83; 124.40; 113.29; 112.30; 78.69; 74.43; 46.75; 23.94; 10.55. Anal. calcd for $C_{30}H_{24}Br_4N_2O$, %: C, 48.16; H, 3.23; N, 3.74. Found, %: C, 48.26; H, 3.24; N, 3.46.

1,3-Bis[3,6-bis(4,4'-dimethoxydiphenylamine)-9*H*-carbazol-9-yl]-2-propoxypropane (2Cz-OMeDPA)

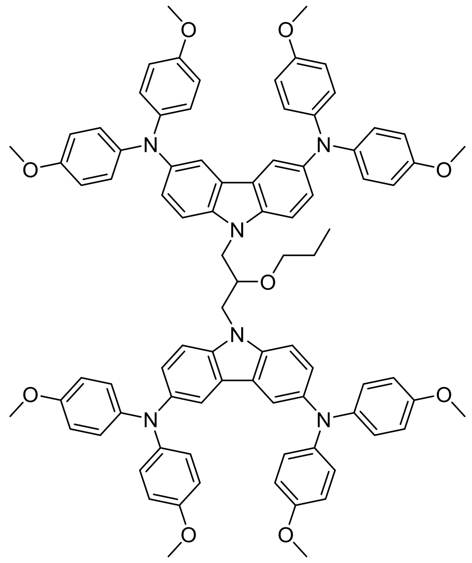

A solution of **4** (1.5 g, 2 mmol) and 4,4′-dimethoxydiphenylamine (2.75 g, 12 mmol) in dry toluene (21.2 mL) was purged with argon for 30 min. Afterwards palladium(II) acetate (0.009 g, 0.04 mmol) and tri-*tert*-butylphosphonium tetrafluoroborate (0.016 g, 0.054 mmol) were added and the mixture was purged with argon for 10 min. Then sodium *tert*-butoxide (1.15 g, 12 mmol) was added and the reaction mixture was heated at reflux under argon atmosphere for 65 h. After completion of the reaction (TLC, tetrahydrofuran:*n*-hexane, 7:18, v:v), reaction mixture was filtered through Celite and extracted with ethyl acetate. The organic layer was dried over anhydrous Na_2SO_4, filtered and the solvent was distilled off under reduced pressure. The crude product was purified by column chromatography (acetone:tetrahydrofuran:*n*-hexane, 5:1:19, v:v:v). 20% solution of the residue in toluene was poured with intensive stirring into a 15-fold excess of *n*-hexane. The precipitate was filtered off, washed with *n*-hexane, and dried. Yield 2.26 g (84.3%). ^{1}H NMR (400 MHz, acetone–d_6) δ, ppm: 7.61 (br s, 4H); 7.39 (d, *J* = 8.8 Hz, 4H); 7.11 (dd, *J* = 8.8, 1.9 Hz, 4H); 6.89 (d, *J* = 8.9 Hz, 16H); 6.75 (d, *J* = 8.9 Hz, 16H); 4.66–4.55 (m, 2H); 4.52–4.41 (m, 2H); 4.34–4.24 (m, 1H); 3.70 (s, 24H); 2.93 (t, *J* = 6.3 Hz, 2H); 1.05 (m, 2H); 0.46 (t, *J* = 7.4 Hz, 3H); ^{13}C NMR (101 MHz, acetone–d_6) δ, ppm: 155.80; 143.44; 141.99; 138.60; 125.19; 124.85; 124.30; 117.16; 115.33; 111.29; 78.88; 73.98; 55.65; 46.38; 23.84; 10.74. FT-IR, ῡ (cm^{-1}): 3037 (aromatic CH); 2993, 2931, 2905, 2832 (aliphatic CH); 1606, 1575, 1500, 1484, 1462, 1440 (C=C); 1235, 1034 (C–O–C). Anal. calcd for

$C_{86}H_{80}N_6O_9$, %: C, 76.99; H, 6.01; N, 6.26. Found, %: C, 77.01; H, 6.18; N, 6.20. $C_{86}H_{80}N_6O_9$[M+] exact mass = 1340.599, MS (MALDI-TOF) = 1340,667.

1,3-Bis(3,6-dibromo-9*H*-carbazol-9-yl)-2-(2,3-epoxy)propoxypropane (6)

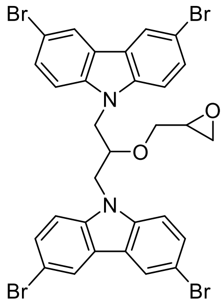

To a solution of **3** (7.06 g, 10 mmol) in tetrahydrofuran (33.4 mL) epichlorohydrin (4.7 ml, 60 mmol) was added. Afterwards anhydrous Na_2SO_4 (0.57 g, 4 mmol) and 85% powdered KOH (1.68 g, 30 mmol) were added and the reaction mixture was stirred at 40 °C for 24 h. After completion of the reaction (TLC, acetone:*n*-hexane, 3:22, v:v), the reaction mixture was extracted with ethyl acetate. The organic layer was dried over anhydrous Na_2SO_4, filtered and the solvent was distilled off under reduced pressure. 20% solution of the residue in tetrahydrofuran was poured with intensive stirring into a 10-fold excess of ethanol. The precipitate was filtered off, washed with ethanol, and dried. The crude product was used for the further synthesis. Yield 6.62 g (86.9%).

^{1}H NMR (400 MHz, tetrahydrofuran–d_8) δ, ppm: 8.30 (br s, 2H); 8.28 (br s, 2H); 7.56–7.46 (m, 6H); 7.38 (d, J = 8.7 Hz, 2H); 4.68–4.38 (m, 4H); 4.37–4.29 (m, 1H); 3.00 (dd, J = 11.8, 3.3 Hz, 1H); 2.77 (dd, J = 11.8, 6.0 Hz, 1H); 2.33–2.27 (m, 1H); 2.23–2.18 (m, 1H); 1.91 (dd, J = 5.1, 2.5 Hz, 1H); ^{13}C NMR (101 MHz, tetrahydrofuran–d_8) δ, ppm: 140.97; 140.87; 130.14; 130.08; 124.88; 124.81; 124.41; 124.39; 113.40; 113.35; 112.38; 112.29; 79.32; 73.78; 50.80; 46.75; 46.71; 44.10. Anal. calcd for $C_{30}H_{22}Br_4N_2O_2$, %: C, 47.28; H, 2.91; N, 3.68. Found, %: C, 46.98; H, 2.99; N, 3.62.

1,6-Bis(3,6-dibromo-9*H*-carbazol-9-yl)-5-(3,6-dibromo-9*H*-carbazol-9-methyl)-4-oxa-2-hexanol (7)

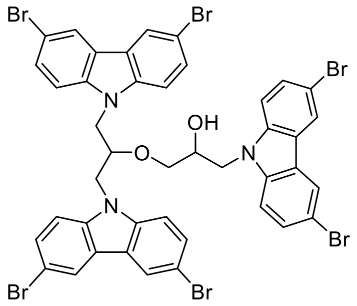

To a solution of **6** (3.81 g, 5 mmol) and 3,6-dibromocarbazole (1.63 g, 5 mmol) in tetrahydrofuran (76.5 mL), anhydrous Na_2SO_4 (0.28 g, 0.2 mmol) and 85% powdered KOH (0.56 g, 10 mmol) were added. The reaction mixture was stirred at 40 °C for 96 h. After completion of the reaction (TLC, acetone:*n*-hexane, 1:4, v:v), the reaction mixture was extracted with ethyl acetate. The organic layer was dried over anhydrous Na_2SO_4, filtered and the solvent was distilled off under reduced pressure. The crude product was purified by column chromatography (tetrahydrofuran:*n*-hexane, 7:18, v:v). 20% solution of the residue in tetrahydrofuran was poured with intensive stirring into a 10-fold excess of ethanol. The precipitate was filtered off, washed with ethanol, and dried. Yield 4.36 g (80.2%).

^{1}H NMR (400 MHz, DMSO–d_6) δ, ppm: 8.46 (br s, 2H); 8.44 (br s, 2H); 8.36 (br s, 2H); 7.87–7.78 (m, 4H,); 7.61 (dd, *J* = 8.8, 1.7 Hz, 2H); 7.57 (dd, *J* = 8.8, 1.7 Hz, 2H); 7.47 (dd, *J* = 8.8, 1.7 Hz, 2H); 6.89 (d, *J* = 8.8 Hz, 2H); 4.81–4.68 (m, 4H); 4.37 (d, *J* = 5.4 Hz, 1H); 4.34–4.26 (m, 1H); 3.39 (dd, *J* = 14.7, 2.6 Hz, 1H); 3.01 (dd, *J* = 14.8, 8.0 Hz, 1H); 2.94–2.85 (m, 1H); 2.44–2.36 (m, 1H); 2.24–2.15 (m, 1H); ^{13}C NMR (101 MHz, DMSO–d_6) δ, ppm: 139.66; 139.57; 139.28; 128.85; 128.48; 123.45; 123.08; 123.03; 123.01; 122.71; 112.27; 112.22; 111.62; 111.57; 111.07; 77.64; 77.34; 73.16; 67.29; 45.45; 45.34; 45.23. Anal. calcd for $C_{42}H_{29}Br_6N_3O_2$, %: C, 46.40; H, 2.69; N, 3.87. Found, %: C, 46.17; H, 2.99; N, 3.63.

1,6-Bis(3,6-dibromo-9*H*-carbazol-9-yl)-5-(3,6-dibromo-9*H*-carbazol-9-methyl)-4-oxa-2-propoxyhexane (7`)

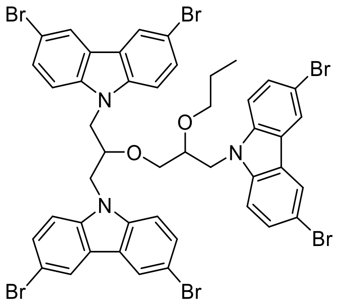

To a solution of **7** (3.26 g, 3 mmol) in tetrahydrofuran (49.5 mL), 1-bromopropane (8.1 mL, 90 mmol) was added. Afterwards anhydrous Na_2SO_4 (0.17 g, 1.2 mmol) and 85% powdered KOH (0.5 g, 9 mmol) were added and the reaction mixture was heated at reflux for 20 h. After completion of the reaction (TLC, acetone:toluene:*n*-hexane, 4:10:11, v:v:v), the reaction mixture was extracted with ethyl acetate. The organic layer was dried over anhydrous Na_2SO_4, filtered and the solvent was distilled off under reduced pressure. The crude product was purified by column chromatography (acetone:*n*-hexane, 3:22, v:v). 20% solution of the crude product in acetone was poured with intensive stirring into a 10-fold excess of ethanol. The precipitate was filtered off, washed with ethanol, and dried. Yield 2.47 g (72.8%).

^{1}H NMR (400 MHz, DMSO–d_6) δ, ppm: 8.43(m, 4H); 8.33 (s, 2H); 7.86 (dd, J = 8.8, 3.0 Hz, 4H); 7.64–7.57 (m, 4H); 7.44 (d, J = 8.8 Hz, 2H); 6.81 (d, J = 8.8 Hz, 2H); 4.84–4.70 (m, 4H); 4.36–4.28 (m, 1H); 3.25–3.15 (m, 2H); 2.61–2.54 (m, 1H); 2.33 (d, J = 4,0 Hz, 2H); 2.17–2.08 (m, 1H); 1.95–1.86 (m, 1H); 0.52–0.37 (m, 2H); 0.06 (t, J = 7.3 Hz, 3H); ^{13}C NMR (101 MHz, DMSO–d_6) δ, ppm: 139.68; 139.55; 138.99; 128.89; 128.82; 128.45; 123.42; 123.09; 123.05; 122.65; 112.27; 111.65; 111.60; 111.53; 111.18; 77.19; 76.36; 70.46; 70.35; 45.31; 45.21; 43.87; 43.38; 21.82; 9.56. Anal. calcd for $C_{45}H_{35}Br_6N_3O_2$, %: C, 47.86; H, 3.12; N, 3.72. Found, %: C, 47.62; H, 3.16; N, 3.72.

1,6-Bis[3,6-bis(4,4'-dimethoxydiphenylamine)-9*H*-carbazol-9-yl]-5-[3,6-bis(4,4'-dimethoxydiphenylamine)-9*H*-carbazol-9-methyl]-4-oxa-2-propoxyhexane (3Cz-OMeDPA)

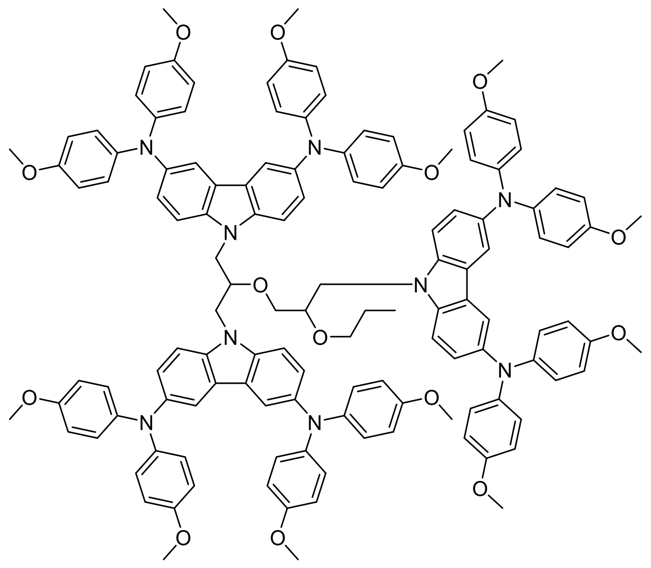

A solution of 1,6-bis(3,6-dibromo-9*H*-carbazol-9-yl)-5-(3,6-dibromo-9*H*-carbazol-9-methyl)-4-oxa-2-propoxyhexane (**7`**) (2.26 g, 2 mmol) and 4,4′-dimethoxydiphenylamine (4.13 g, 18 mmol) in dry toluene (31.9 mL) was purged with argon for 30 min. Afterwards palladium(II) acetate (0.009 g, 0.04 mmol) and tri-*tert*-butylphosphonium tetrafluoroborate (0.016 g, 0.054 mmol) were added and the reaction mixture was purged with argon for 10 min. Then sodium *tert*-butoxide (1.15 g, 12 mmol) was added and the reaction mixture was heated at reflux under argon atmosphere for 144 h. After completion of the reaction (TLC, acetone:*n*-hexane, 7:18, v:v), reaction mixture was filtered through Celite and extracted with ethyl acetate. The organic layer was dried over anhydrous Na_2SO_4, filtered and the solvent was distilled off under reduced pressure. The crude product was purified by column chromatography (acetone:tetrahydrofuran:*n*-hexane, 5:2:18, v:v:v). 20% solution of the crude product in toluene was poured with intensive stirring into a 15-fold excess of *n*-hexane. The precipitate was filtered off, washed with *n*-hexane, and dried. Yield 2.28 g (56.5%).

^{1}H NMR (400 MHz, acetone–d_6) δ, ppm: 7.68 (d, J = 8.8 Hz, 2H); 7.64 (d, J = 8.8 Hz, 2H); 7.55 (br s, 3H); 7.50 (br s, 3H); 7.13–7.02 (m, 8H); 6.90–6.53 (m, 48H); 4.89–4.74 (m, 4H); 4.64–4.56 (m, 1H); 3.74-3.50 (m, 36H); 3.30–3.16 (m, 1H); 2.55–2.30 (m, 5H); 2.15–2.07 (m, 1H); 0.78 (m, 2H); 0.26 (t, J = 7.4 Hz, 3H); ^{13}C NMR (101 MHz, acetone–d_6) δ, ppm: 155.76; 155.68; 155.60; 143.42; 143.35; 141.94; 141.78; 138.90; 125.70; 125.12; 124.94; 124.84; 124.34; 124.11; 123.88; 117.87; 117.21; 115.27; 115.26; 111.72;

111.55; 78.72; 77.90; 72.18; 71.43; 55.66; 55.64; 55.63; 46.08; 45.99; 45.95; 23.59; 10.59; FT-IR, ῡ (cm^{-1}): 3038 (aromatic CH); 2994, 2931, 2904, 2832 (aliphatic CH); 1606, 1575, 1499, 1483, 1461, 1440 (C=C); 1233, 1033 (C–O–C). Anal. calcd for $C_{129}H_{119}N_9O_{14}$, %: C, 76.73; H, 5.94; N, 6.24. Found, %: C, 76.81; H, 6.14; N, 6.10. $C_{129}H_{119}N_9O_{14}$[M+] exact mass = 2017.888, MS (MALDI-TOF) = 2018.145.

9-Benzyl-3,6-dibromo-9*H*-carbazole (8)

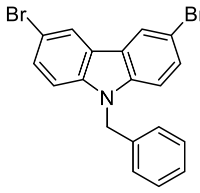

To a solution of 3,6-dibromocarbazole (6.5 g, 20 mmol) in toluene (32.5 mL), catalytic amount of interphase catalyst tetrabutylammonium bromide and KOH (18.52 g, 330 mmol) were added followed by distilled water (32.5 mL) and benzyl bromide (3.56 mL, 30 mmol). The reaction mixture was heated at reflux for 6 h. After completion of the reaction (TLC, acetone:*n*-hexane, 1:4, v:v), the reaction mixture was extracted with ethyl acetate. The organic layer was dried over anhydrous Na_2SO_4, filtered and the solvent was distilled off under reduced pressure. The crude product was crystallized from tetrahydrofuran/ethanol 2:3 mixture (60 mL). The precipitate was filtered off, washed with ethanol, and dried. Yield 7.69 g (92.6%).

^{1}H NMR and ^{13}C NMR spectra, elemental analysis data, and melting point for compound **8** were found to be identical with those described in [1].

9-Benzyl-3,6-bis(4,4'-dimethoxydiphenylamine)-9*H*-carbazole (9)

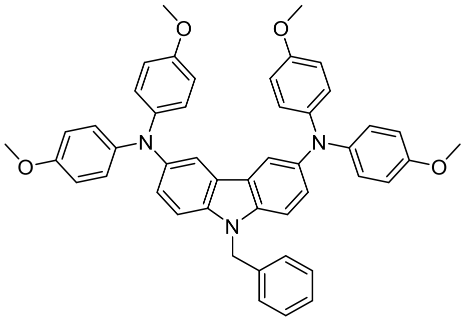

A solution of **8** (4.98 g, 12 mmol) and 4,4′-dimethoxydiphenylamine (8.25 g, 36 mmol) in dry toluene (67 mL) was purged with argon for 30 min. Afterwards palladium(II) acetate (0.054 g, 0.24 mmol) and tri-*tert*-

butylphosphonium tetrafluoroborate (0.094 g, 0.324 mmol) were added and the mixture was purged with argon for 10 min. Then sodium *tert*-butoxide (6.92 g, 72 mmol) was added and the reaction mixture was heated at reflux under argon atmosphere for 20 h. After completion of the reaction (TLC, acetone:*n*-hexane, 1:4, v:v), reaction mixture was filtered through Celite and extracted with ethyl acetate. The organic layer was dried over anhydrous Na_2SO_4, filtered and the solvent was distilled off under reduced pressure. The crude product was purified by column chromatography (acetone:tetrahydrofuran:*n*-hexane, 3:5:42, v:v:v). A 20% solution of the resulting product in acetone was poured with intensive stirring into a 7.5-fold excess of ethanol. The precipitate was filtered off, washed with ethanol, and dried. Yield 7.99 g (93.5%).

^{1}H NMR and ^{13}C NMR spectra, elemental analysis data, and melting point for compound **9** were found to be identical with those described in [1].

3,6-Bis(4,4'-dimethoxydiphenylamine)-9*H*-carbazole (10)

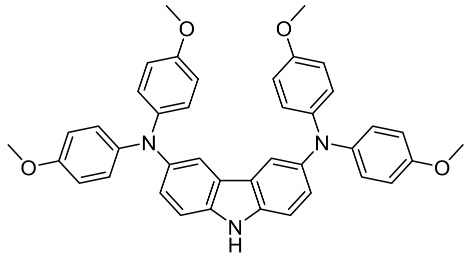

To a solution of **9** (3 g, 4.2 mmol) in dimethyl sulfoxide (15 mL) a solution of potassium *tert*-butoxide (4.73 g, 42.1 mmol)) in tetrahydrofuran (42.1 mL) was added. The reaction mixture was stirred at room temperature for 4 h in an open flask to allow air oxygen enter the reaction mixture. After completion of the reaction (TLC, acetone:*n*-hexane, 1:4, v:v), distilled water (300 mL) was poured into reaction mixture, precipitate formed was filtered off, washed with distilled water until neutral medium, washed with ethanol, and dried. Yield 2.5 g (95.4%).

^{1}H NMR and ^{13}C NMR spectra, elemental analysis data, and melting point for compound **10** were found to be identical with those described in [1].

9-(2,3-epoxy)propyl-3,6-bis(4,4'-dimethoxydiphenylamine)-9*H*-carbazole (11)

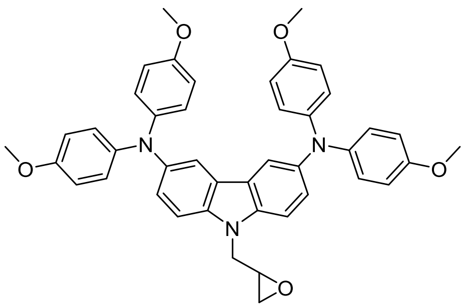

To a solution of **10** (3.11 g, 5 mmol) in epichlorohydrin (37.3 mL), anhydrous Na_2SO_4 (0.28 g, 2 mmol) and 85% powdered KOH (0.84 g, 15 mmol) were added and the reaction mixture was stirred at 40 °C for 22 h. After completion of the reaction (TLC, tetrahydrofuran:*n*-hexane, 7:18, v:v), the reaction mixture was filtered off to separate the precipitate of polymerized epichlorohydrin and the filtrate was extracted with ethyl acetate. The organic layer was dried over anhydrous Na_2SO_4, filtered and the solvent was distilled off under reduced pressure. The crude product was purified by column chromatography (tetrahydrofuran:*n*-hexane, 4:21, v:v). 20% solution of the resulting product in acetone was poured with intensive stirring into a 12.5-fold excess of ethanol. The precipitate was filtered off, washed with ethanol, and dried. Yield 2.86 g (84.5%).

^{1}H NMR (400 MHz, acetone–d_6) δ, ppm: 7.61 (br s, 2H); 7.51 (d, *J* = 8.8 Hz, 2H); 7.15 (dd, *J* = 8.7, 1.7 Hz, 2H); 6.90 (d, *J* = 8.9 Hz, 8H); 6.78 (d, *J* = 8.9 Hz, 8H); 4.74 (dd, *J* = 15.8, 2.6 Hz, 1H); 4.32 (dd, *J* = 15.8, 5.7 Hz, 1H); 3.72 (s, 12H); 3.35–3.29 (m, 1H); 2.79–2.74 (m, 1H); 2.63 (dd, *J* = 4.8, 2.4 Hz, 1H); ^{13}C NMR (101 MHz, acetone–d_6) δ, ppm: 155.79; 143.47; 142.09; 138.74; 125.22; 124.97; 124.25; 117.14; 115.33; 111.14; 55.65; 51.25; 45.80; 45.35. Anal. calcd for $C_{43}H_{39}N_3O_5$, %: C, 76.20; H, 5.80; N, 6.20. Found, %: C, 75.96; H, 5.76; N, 6.19.

1,3-Bis[3,6-bis(4,4'-dimethoxydiphenylamine)-9*H*-carbazol-9-yl]-2-propanol (2Cz-OMeDPA-OH)

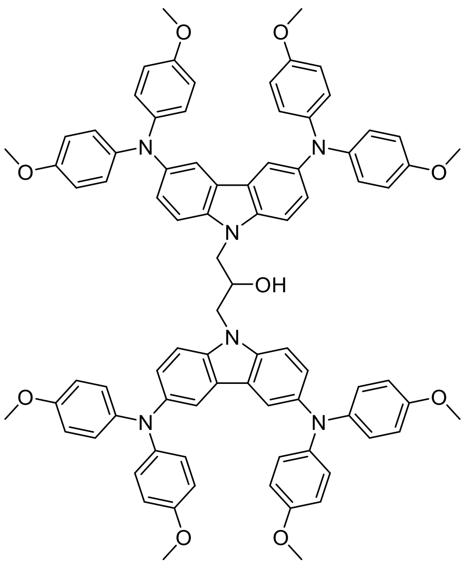

To a solution of **11** (2.71 g, 4 mmol) and **10** (2.49 g, 4 mmol) in tetrahydrofuran (17.7 mL), anhydrous Na_2SO_4 (0.23 g, 1.6 mmol) and 85% powdered KOH (0.45 g, 8 mmol) were added and the reaction mixture was stirred at 40 °C for 31 h. After completion of the reaction (TLC, tetrahydrofuran:*n*-hexane, 7:18, v:v), the reaction mixture was extracted with ethyl acetate. The organic layer was dried over anhydrous Na_2SO_4, filtered and the solvent was distilled off under reduced pressure. The crude product was purified by column chromatography (acetone:*n*-hexane, 13:37, v:v). 20% solution of the resulting product in acetone was poured with intensive stirring into an 8.75-fold excess of ethanol. The precipitate was filtered off, washed with ethanol, and dried. Yield 4.02 g (77.4%).

^{1}H NMR (400 MHz, acetone–d_6) δ, ppm: 7.60 (br s, 4H); 7.37 (d, *J* = 8.8 Hz, 4H); 7.10 (dd, *J* = 8.7, 1.7 Hz, 4H); 6.88 (d, *J* = 8.9 Hz, 16H); 6.74 (d, *J* = 8.9 Hz, 16H); 4.82–4.69 (m, 2H); 4.58–4.38 (m, 4H); 3.70 (s, 24H); ^{13}C NMR (101 MHz, acetone–d_6) δ, ppm: 155.76; 143.46; 141.90; 138.79; 125.15; 124.93; 124.27; 117.23; 115.32; 111.24; 70.36; 55.65); 48.27; FT-IR, $\bar{\upsilon}$ (cm^{-1}): 3484 (OH); 3038 (aromatic CH); 2997, 2931, 2905, 2832 (aliphatic CH); 1606, 1575, 1499, 1483, 1462, 1440 (C=C); 1232, 1033 (C–O–C). Anal. calcd for $C_{83}H_{74}N_6O_9$, %: C, 76.71; H, 5.74; N, 6.47. Found, %: C, 76.59; H, 5.63; N, 6.41. $C_{83}H_{74}N_6O_9$[M+] exact mass = 1298.552, MS (MALDI-TOF) = 1298.606.

2-(2,3-epoxy)propoxy-1,3-bis[3,6-bis(4,4'-dimethoxydiphenylamine)-9*H*-carbazol-9-yl]propane (12)

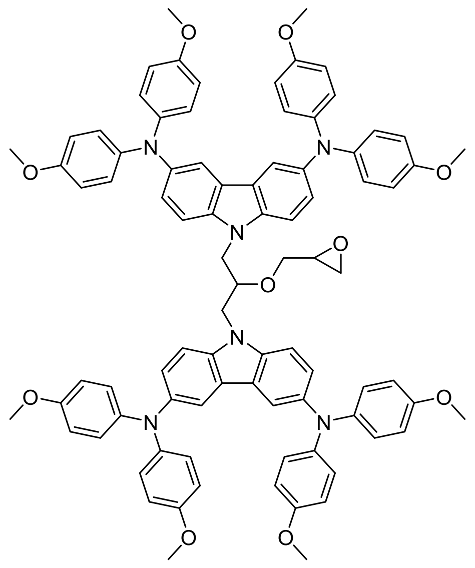

To a solution of **2Cz-OMeDPA-OH** (3.25 g, 2.5 mmol) in epichlorohydrin (17.4 mL), anhydrous Na_2SO_4 (0.14 g, 1 mmol) and 85% powdered KOH (0.42 g, 7.5 mmol) were added and the reaction mixture was stirred at 40 °C for 53 h. After completion of the reaction (TLC, acetone:toluene:*n*-hexane, 1:2:2, v:v:v), the reaction mixture was filtered off to separate the precipitate of polymerized epichlorohydrin, and then filtrate was extracted with ethyl acetate. The organic layer was dried over anhydrous Na_2SO_4, filtered and the solvent was distilled off under reduced pressure. The crude product was purified by column chromatography (tetrahydrofuran:toluene:*n*-hexane, 4:8:13, v:v:v). A 20% solution of the resulting product in acetone was poured with intensive stirring into a 10-fold excess of ethanol. The precipitate was filtered off, washed with ethanol, and dried. Yield 2.85 g (84.0%).

^{1}H NMR (400 MHz, DMSO–d_6) δ, ppm: 7.64 (s, 4H); 7.50 (d, J = 8.8 Hz, 2H); 7.44 (d, J = 8.7 Hz, 2H); 7.07 (d, J = 8.1 Hz, 4H); 6.83 (d, J = 8.8 Hz, 16H); 6.76 (d, J = 8.8 Hz, 16H); 4.65–4.36 (m, 4H); 4.30–4.19 (m, 1H); 3.66 (s, 24H); 2.89 (d, J = 4.3 Hz, 2H); 2.32–2.26 (m, 1H); 2.26–2.21 (m, 1H); 1.91 (dd, J = 4.0, 2.0 Hz, 1H); ^{13}C NMR (101 MHz, DMSO–d_6) δ, ppm: 154.17; 142.02; 140.22; 140.20; 137.50; 137.44; 124.25; 123.68; 122.75; 116.78; 114.61; 110.75; 78.15; 71.68; 55.13; 49.56; 45.10; 43.50. Anal. calcd for $C_{86}H_{78}N_6O_{10}$, %: C, 76.20; H, 5.80; N, 6.20. Found, %: C, 76.09; H, 5.74; N, 6.13.

1,6-Bis[3,6-bis(4,4'-dimethoxydiphenylamine)-9*H*-carbazol-9-yl]-5-[3,6-bis(4,4'-dimethoxydiphenylamine)-9*H*-carbazol-9-methyl]-4-oxa-2-hexanol (3Cz-OMeDPA-OH)

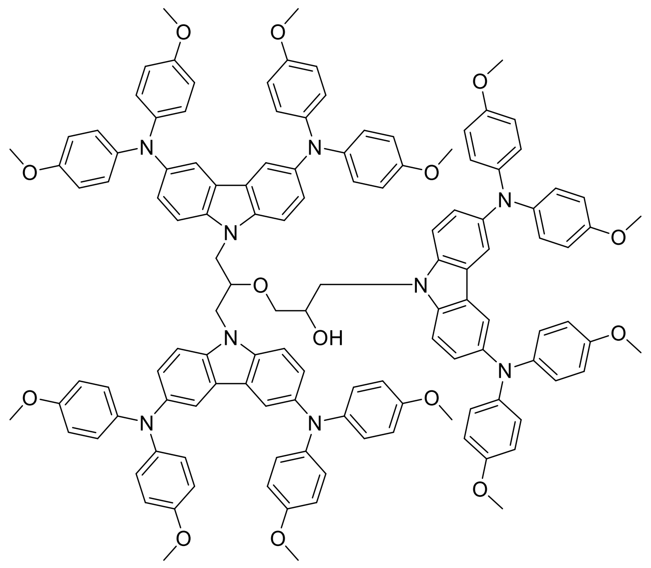

To a solution of **11** (2.71 g, 2 mmol) and **10** (1.24 g, 2 mmol) in tetrahydrofuran (15.6 ml), anhydrous Na_2SO_4 (0.11 g, 0.8 mmol) and 85% powdered KOH (0.22 g, 4 mmol) were added and the reaction mixture was stirred at 40 °C for 43 h. After completion of the reaction (TLC, acetone:*n*-hexane, 7:18, v:v), the reaction mixture was extracted with ethyl acetate. The organic layer was dried over anhydrous Na_2SO_4, filtered and the solvent was distilled off under reduced pressure. The crude product was purified by column chromatography (acetone:*n*-hexane, 7:18, v:v). A 20% solution of the resulting product in acetone was poured with intensive stirring into a 10-fold excess of ethanol. The precipitate was filtered off, washed with ethanol, and dried. Yield 2.8 g (70.8%).

^{1}H NMR (400 MHz, acetone–d_6) δ, ppm: 7.60–7.47 (m, 10H); 7.12–7.04 (m, 4H); 7.04–6.95 (m, 4H); 6.86–6.74 (m, 24H); 6.72–6.59 (m, 24H); 4.85–4.52 (m, 5H); 3.80-3.73, (m, 1H); 3.70-3.63 (m, 36H); 3.59–3.50 (m, 3H); 3.08–2.98 (m, 2H); ^{13}C NMR (101 MHz, acetone–d_6) δ, ppm: 155.73; 155.69; 155.65; 143.46; 143.37; 143.33; 142.10; 141.99; 141.75; 138.86; 138.74; 138.64; 125.28; 125.21; 125.06; 124.98; 124.61; 124.39; 124.35; 123.97; 117.51; 117.18; 116.58; 115.30; 115.28; 111.40; 111.36; 111.32; 78.84; 73.70; 69.93; 55.64; 47.08; 46.22; 45.98; FT-IR, $\bar{\upsilon}$ (cm^{-1}): 3540 (OH); 3039 (aromatic CH); 2988, 2931, 2905, 2832 (aliphatic CH); 1606, 1574, 1500, 1484, 1462, 1440 (C=C); 1235, 1034 (C–O–C). Anal. calcd for $C_{126}H_{113}N_9O_{14}$, %: C, 76.54; H, 5.76; N, 6.38. Found, %: C, 76.45; H, 5.83; N, 6.19. $C_{126}H_{113}N_9O_{14}$[M+] exact mass = 1975.841, MS (MALDI-TOF) = 1976.181.

2-(2,3-epoxy)propoxy-1,6-bis[3,6-bis(4,4'-dimethoxydiphenylamine)-9*H*-carbazol-9-yl]-5-[3,6-bis(4,4'-dimethoxydiphenylamine)-9*H*-carbazol-9-methyl]-4-oxahexane (13)

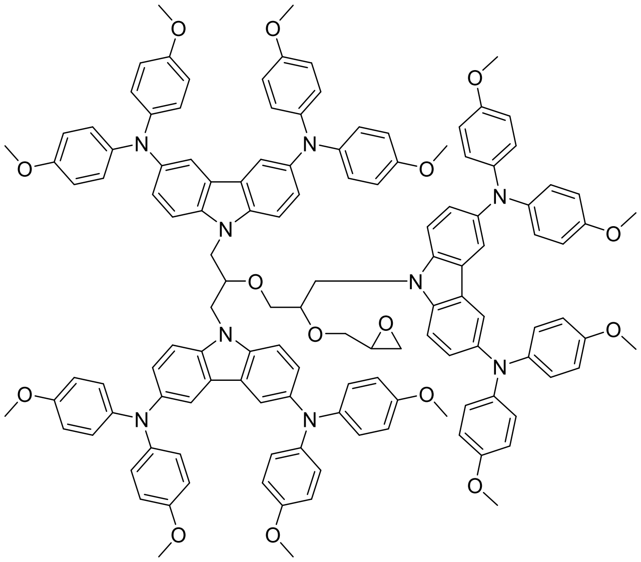

To a solution of **3Cz-OMeDPA-OH** (2.18 g, 1.1 mmol) in epichlorohydrin (11.1 mL), anhydrous Na_2SO_4 (0.06 g, 0.44 mmol) and 85% powdered KOH (0.19 g, 3.3 mmol) were added and the reaction mixture was stirred at 40 °C for 168 h. After completion of the reaction (TLC, acetone:toluene:*n*-hexane, 5:8:12, v:v:v), the reaction mixture was filtered off to separate the precipitate of polymerized epichlorohydrin, and then filtrate was extracted with ethyl acetate. The organic layer was dried over anhydrous Na_2SO_4, filtered and the solvent was distilled off under reduced pressure. The crude product was purified by column chromatography (acetone:toluene:*n*-hexane, 4:8:13, v:v:v). A 20% solution of the resulting product in acetone was poured with intensive stirring into a 10-fold excess of ethanol. The precipitate was filtered off, washed with ethanol, and dried. Yield 1.63 g (72.8%).

^{1}H NMR (400 MHz, acetone–d_6) δ, ppm: 7.72–7.48 (m, 10H); 7.13–7.00 (m, 8H); 6.90–6.58 (m, 48H); 4.90–4.76 (m, 4H); 4.65–4.56 (m, 1H); 3.70–3.63 (m, 36H); 3.61–3.51 (m, 2H); 3.36–3.27 (m, 1H); 2.64–2.36 (m, 4H); 2.32–2.22 (m, 2H); 1.95 (dd, *J* = 5.3, 2.6 Hz, 1H); 1.82 (dd, *J* = 5.3, 2.5 Hz, 1H); ^{13}C NMR (101 MHz, acetone–d_6) δ, ppm: 155.82; 155.80; 155.69; 155.65; 143.43; 143.41; 143.37; 141.95; 141.92; 138.94; 138.91; 138.37; 138.34; 125.56; 125.53; 125.11; 124.98; 124.93; 124.91; 124.35; 124.31; 124.15; 123.95; 117.25; 115.34; 115.31; 115.27; 111.76; 111.72; 111.63; 111.59; 72.36; 71.06; 69.18; 59.14; 59.11; 55.65; 55.64; 51.95; 50.65; 47.77; 47.75; 47.69; 44.00; 43.95. Anal. calcd for $C_{129}H_{117}N_9O_{15}$, %: C, 76.20; H, 5.80; N, 6.20. Found, %: C, 76.05; H, 5.89; N, 6.31.

1,9-Bis[3,6-bis(4,4'-dimethoxydiphenylamine)-9*H*-carbazol-9-yl]-5,8-bis[3,6-bis(4,4'-dimethoxydiphenylamine)-9*H*-carbazol-9-methyl]-4,7-dioxa-2-nonanol (4Cz-OMeDPA-OH)

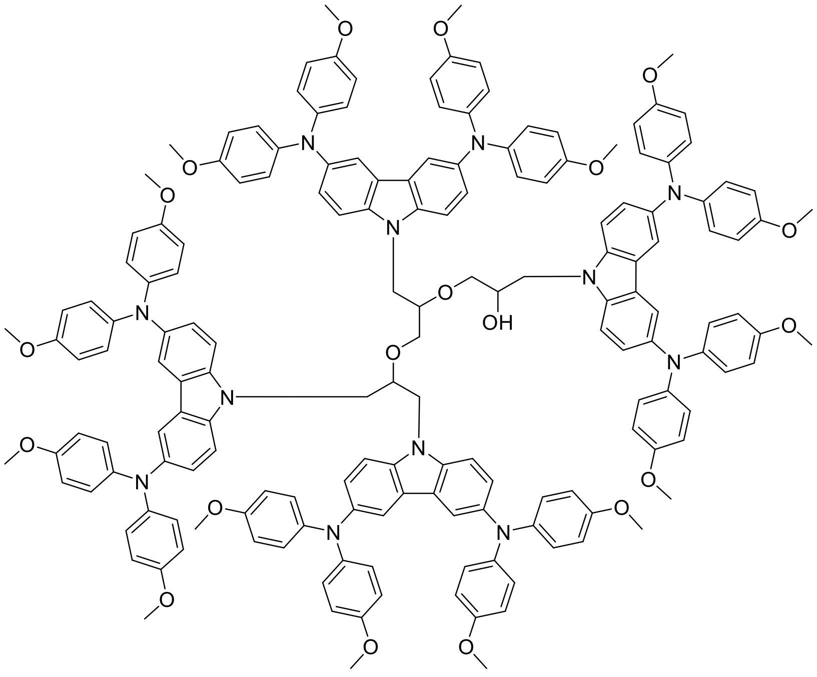

To a solution of **13** (1.42 g, 0.7 mmol) and **10** (0.44 g, 0.7 mmol) in tetrahydrofuran (10.9 mL), anhydrous Na_2SO_4 (0.04 g, 0.28 mmol) and 85% powdered KOH (0.08 g, 1.4 mmol) were added and the reaction mixture was stirred at 40 °C for 53 h. After completion of the reaction (TLC, acetone:*n*-hexane, 8:17, v:v), the reaction mixture was extracted with ethyl acetate. The organic layer was dried over anhydrous Na_2SO_4, filtered and the solvent was distilled off under reduced pressure. The crude product was purified by column chromatography (acetone:*n*-hexane, 7:18, v:v). A 20% solution of the resulting product in acetone was poured with intensive stirring into a 10-fold excess of ethanol. The precipitate was filtered off, washed with ethanol, and dried. Yield 1.27 g (68.2%).

^{1}H NMR (400 MHz, tetrahydrofuran–d_8) δ, ppm: 7.62–7.43 (m, 10H); 7.19–7.09 (m, 8H); 7.03 (dd, *J* = 8.7, 1.3 Hz, 2H); 6.97–6.84 (m, 4H); 6.84–6.70 (m, 25H); 6.67–6.49 (m, 39H); 4.75–4.52 (m, 5H); 3.86–3.68 (m, 4H); 3.68-3.55 (m, 48H, overlaps with THF–d_8); 3.29–3.23 (m, 1H); 3.20–3.14 (m, 1H); 3.14–3.08 (m, 1H); 3.02 (d, *J* = 4.8 Hz, 2H); 2.69–2.63 (m, 1H); ^{13}C NMR (101 MHz, tetrahydrofuran–d_8) δ, ppm: 155.87; 155.83; 155.75; 155.71; 143.68; 143.57; 143.53; 143.50; 142.26; 142.24; 142.22; 142.00; 141.76; 141.69; 141.67; 141.65; 138.80; 138.71; 125.56; 125.54; 125.20; 125.12; 125.05; 125.03; 124.96; 124.67; 124.37; 124.36; 124.30; 124.25; 124.22; 117.95; 117.80; 117.58; 117.34; 117.33; 115.19; 115.16; 111.37; 111.32;

111.30; 110.91; 110.88; 110.84; 81.48; 79.17; 78.98; 78.92; 78.89; 72.54; 71.07; 71.04; 69.72; 69.69; 55.57; 55.54; 50.79; 50.49; 47.62; 46.44; 46.42; 46.33; 46.29; 46.26; 46.22; 45.74; 45.69; FT-IR, ῡ (cm^{-1}): 3541 (OH); 3038 (aromatic CH); 2994, 2930, 2904, 2831 (aliphatic CH); 1606, 1575, 1499, 1483, 1461, 1439 (C=C); 1231, 1032 (C–O–C). Anal. calcd for $C_{169}H_{152}N_{12}O_{19}$, %: C, 76.45; H, 5.77; N, 6.33. Found, %: C, 76.51; H, 5.75; N, 6.37. $C_{169}H_{152}N_{12}O_{19}$[M+] exact mass = 2653.130, MS (MALDI-TOF) = 2654.209.

1,9-Bis[3,6-bis(4,4'-dimethoxydiphenylamine)-9*H*-carbazol-9-yl]-5,8-bis[3,6-bis(4,4'-dimethoxydiphenylamine)-9*H*-carbazol-9-methyl]-4,7-dioxa-2-propoxynonane (4Cz-OMeDPA)

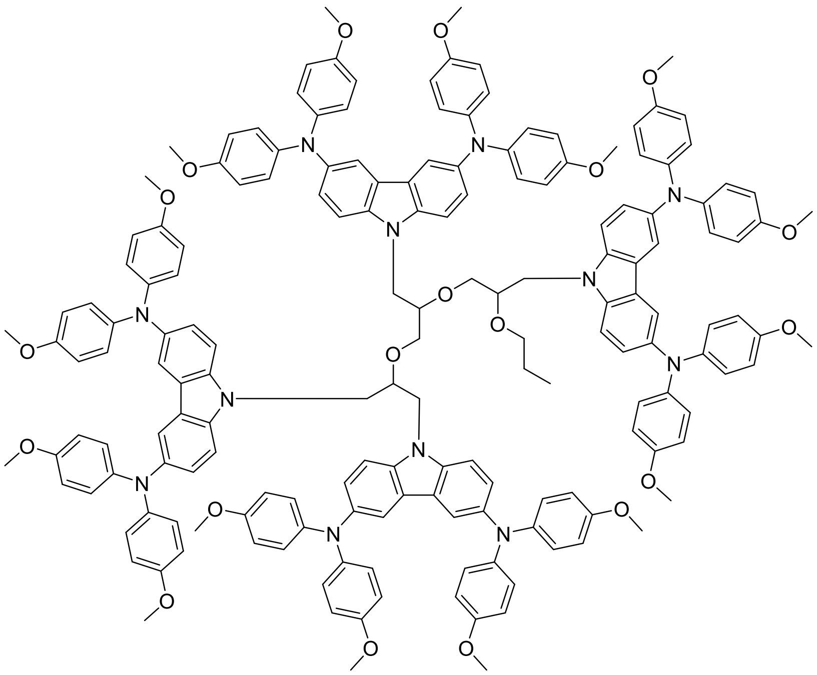

To a solution of **4Cz-OMeDPA-OH** (0.8 g, 0.3 mmol) in tetrahydrofuran (5.7 mL), 1-bromopropane (0.8 mL, 9 mmol) was added. Afterwards anhydrous Na_2SO_4 (0,02 g, 0.12 mmol) and 85% powdered KOH (0.05 g, 0.9 mmol) were added and the reaction mixture was heated at reflux for 45 h. After completion of the reaction (TLC, acetone:toluene:*n*-hexane, 5:8:12, v:v:v), the reaction mixture was extracted with ethyl acetate. The organic layer was dried over anhydrous Na_2SO_4, filtered and the solvent was distilled off under reduced pressure. The crude product was purified by column chromatography (acetone:*n*-hexane, 8:17, v:v). A 20% solution of the resulting product in acetone was poured with intensive stirring into a 14-fold excess of ethanol. The precipitate was filtered off, washed with ethanol, and dried. Yield 0.55 g (67.8%).

^{1}H NMR (400 MHz, acetone–d_6) δ, ppm: 7.77–7.69 (m, 4H); 7.56–7.43 (m, 8H); 7.19–6.92 (m, 12H); 6.89–6.49 (m, 64H); 4.92–4.81 (m, 4H); 4.70–4.62 (m, 1H); 3.77–3.70 (m, 2H); 3.70-3.56 (m, 48H); 3.47–3.37

(m, 2H); 3.08–3.02 (m, 1H); 2.99–2.92 (m, 1H); 2.70–2.59 (m, 4H); 2.50–2.28 (m, 2H); 0.98–0.81 (m, 2H); 0.37–0.26 (m, 3H); ^{13}C NMR (101 MHz, acetone–d_6) δ, ppm: 155.68; 155.66; 155.62; 155.56; 143.47; 143.39; 143.34; 143.04; 142.99; 142.75; 142.73; 142.06; 141.99; 141.97; 141.91; 141.79; 141.75; 141.68; 141.66; 138.87; 138.84; 138.65; 138.51; 138.41; 125.30; 125.22; 125.15; 125.12; 125.08; 124.97; 124.61; 124.33; 124.28; 124.27; 124.02; 123.96; 123.92; 117.23; 117.21; 116.67; 116.63; 115.29; 115.25; 111.57; 111.52; 78.74; 77.62; 72.57; 72.50; 71.23; 70.47; 64.32; 64.26; 61.89; 55.65; 55.62; 53.10; 51.64; 51.61; 51.03; 50.94; 23.64; 10.77; 10.75; FT-IR, ῡ (cm^{-1}): 3038 (aromatic CH); 2994, 2930, 2904, 2832 (aliphatic CH); 1606, 1575, 1499, 1483, 1461, 1439 (C=C); 1232, 1032 (C–O–C). Anal. calcd for $C_{172}H_{158}N_{12}O_{19}$, %: C, 76.59; H, 5.90; N, 6.23. Found, %: C, 76.69; H, 5.86; N, 6.18. $C_{172}H_{158}N_{12}O_{19}$[M+] exact mass = 2695.177, MS (MALDI-TOF) = 2696.270.

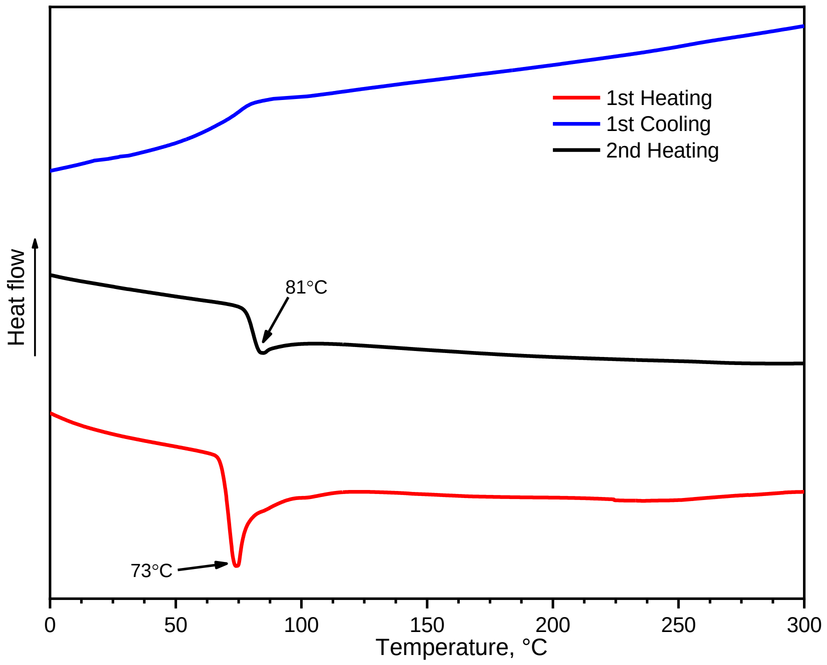

Figure S1. Differential scanning calorimetry (DSC) first and second heating curves of **Cz–OMeDPA** (scan rate 10 ºC/min, N_2 atmosphere).

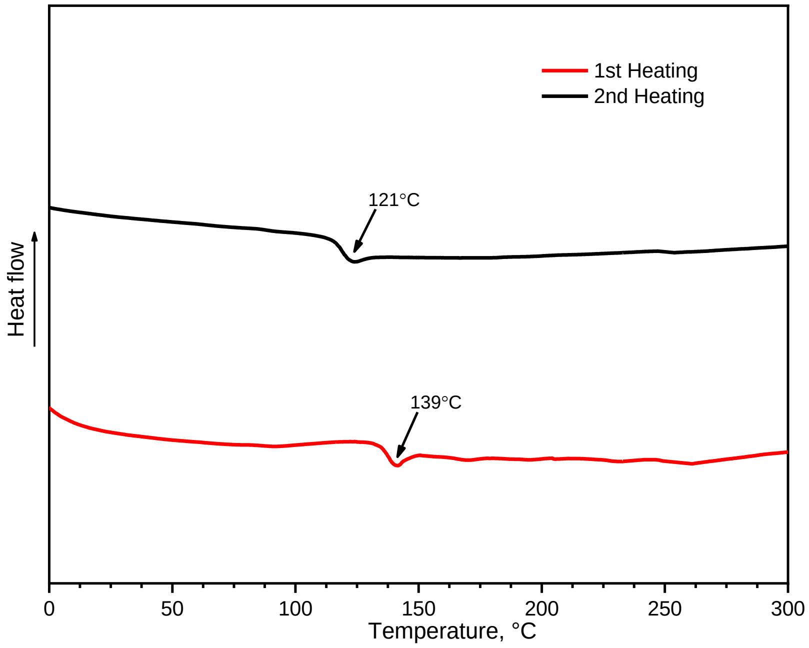

Figure S2. Differential scanning calorimetry (DSC) first and second heating curves of **2Cz–OMeDPA-OH** (scan rate 10 ºC/min, N_2 atmosphere).

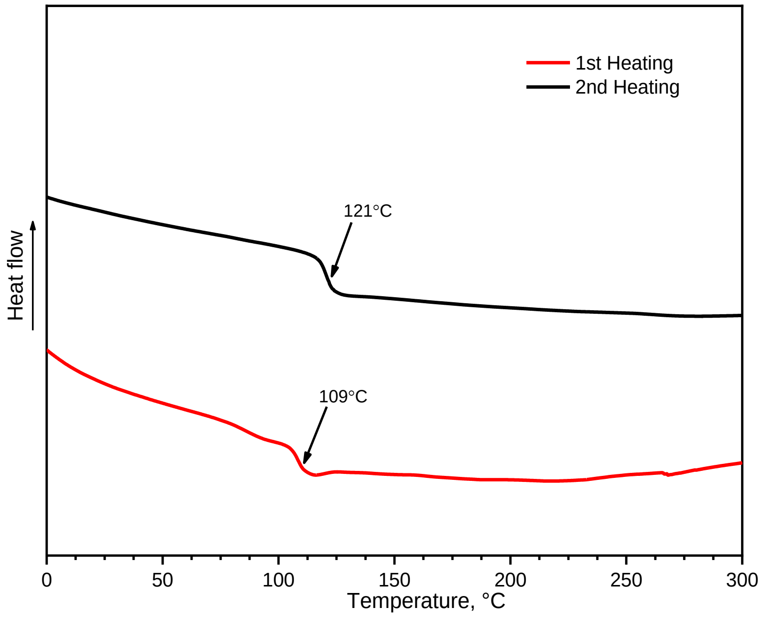

Figure S3. Differential scanning calorimetry (DSC) first and second heating curves of **2Cz–OMeDPA** (scan rate 10 ºC/min, N_2 atmosphere).

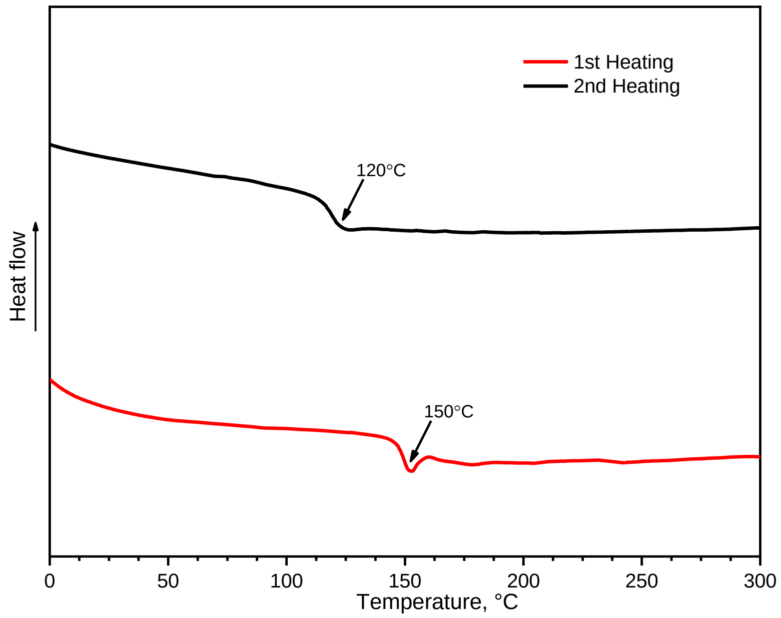

Figure S4. Differential scanning calorimetry (DSC) first and second heating curves of **3Cz–OMeDPA-OH** (scan rate 10 ºC/min, N_2 atmosphere).

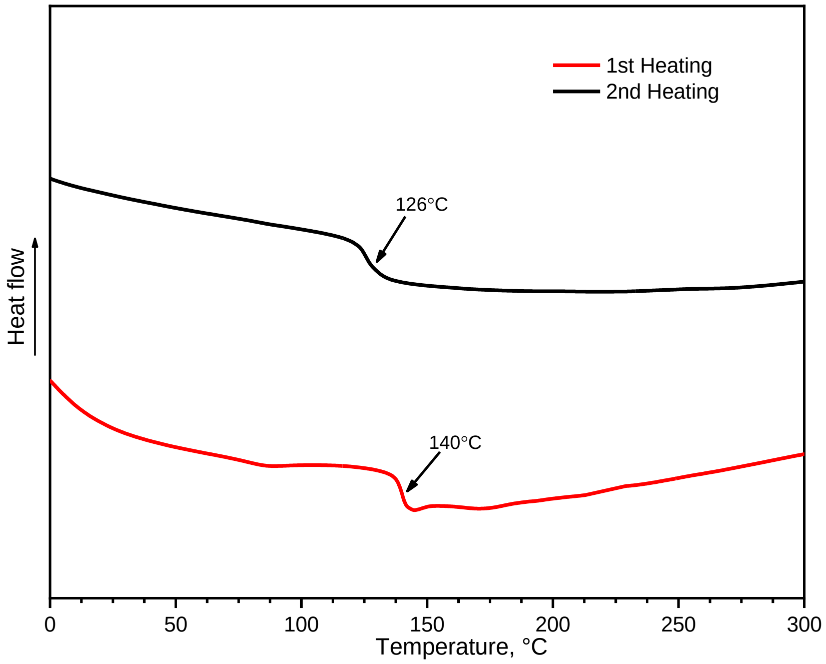

Figure S5. Differential scanning calorimetry (DSC) first and second heating curves of **3Cz–OMeDPA** (scan rate 10 ºC/min, N_2 atmosphere).

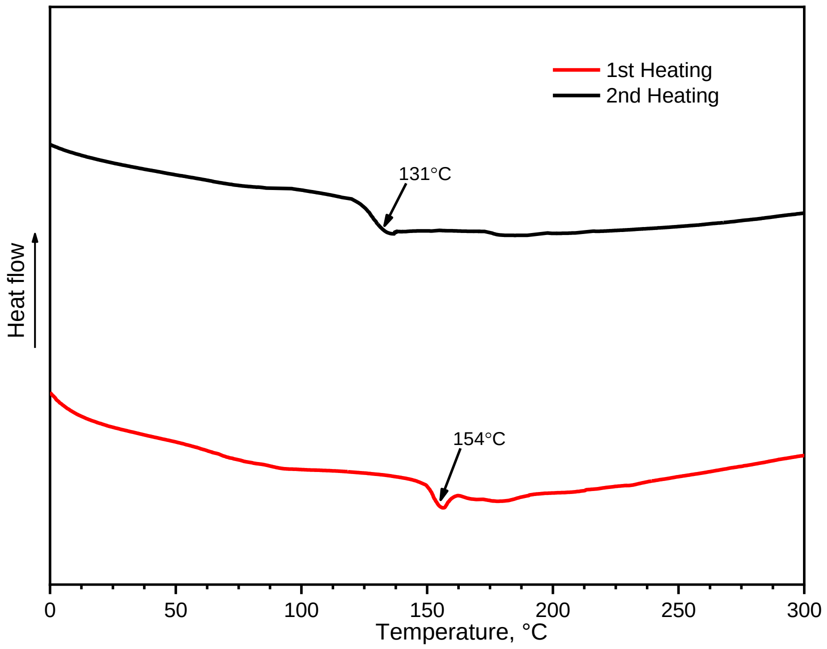

Figure S6. Differential scanning calorimetry (DSC) first and second heating curves of **4Cz–OMeDPA-OH** (scan rate 10 °C/min, N_2 atmosphere).

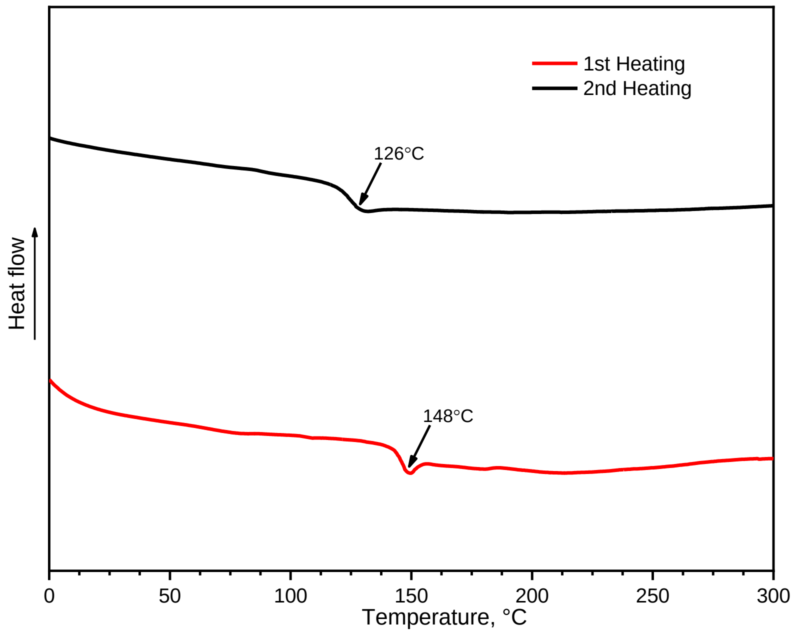

Figure S7. Differential scanning calorimetry (DSC) first and second heating curves of **4Cz–OMeDPA** (scan rate 10 °C/min, N_2 atmosphere).

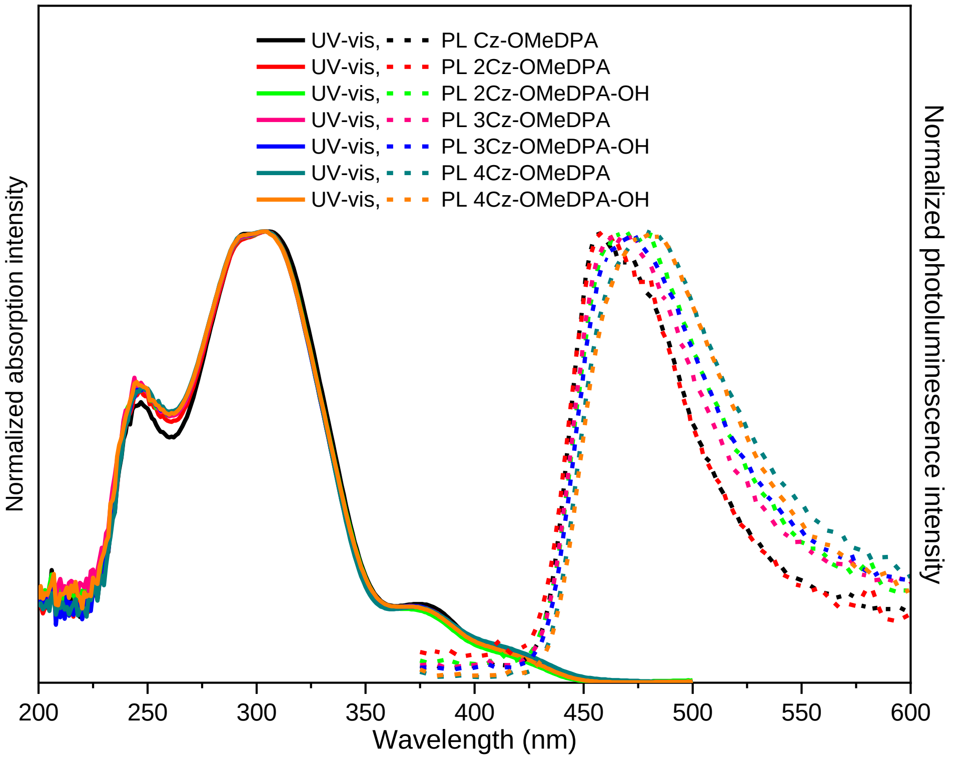

Figure S8. UV−Vis absorption and FL spectra of thin films novel HTMs.

Table S1. Fitting parameters of *bi*-exponential decay function in TRPL of Glass/perovskite substrates based on different HTMs.

Films	Fraction A_1	τ_1 (ns)	Fraction A_2	τ_2 (ns)	Average decay time τ (ns)[a]
Glass/perovskite/spiro-OMeTAD	0.63	16.8	0.37	119.1	99.2
Glass/perovskite/**3Cz–OMeDPA**	0.63	50.7	0.37	357.3	297.6
Glass/perovskite/**2Cz–OMeDPA**	0.59	21.3	0.41	232.4	207.8
Glass/perovskite/**Cz–OMeDPA**	0.63	84.9	0.37	603.2	502.9
Glass/perovskite/**2Cz–OMeDPA–OH**	0.66	63.1	0.34	470.9	361.5
Glass/perovskite/**3Cz–OMeDPA–OH**	0.63	43.6	0.37	263.4	215.1
Glass/perovskite/**4Cz–OMeDPA–OH**	0.63	72.7	0.37	526.9	440.6
Glass/perovskite/**4Cz–OMeDPA**	0.66	143.4	0.34	939.8	757.8

[a]Average decay time is calculated according to the equation: $\tau = (A_1\tau_1^2 + A_2\tau_2^2)/(A_1\tau_1 + A_2\tau_2)$.

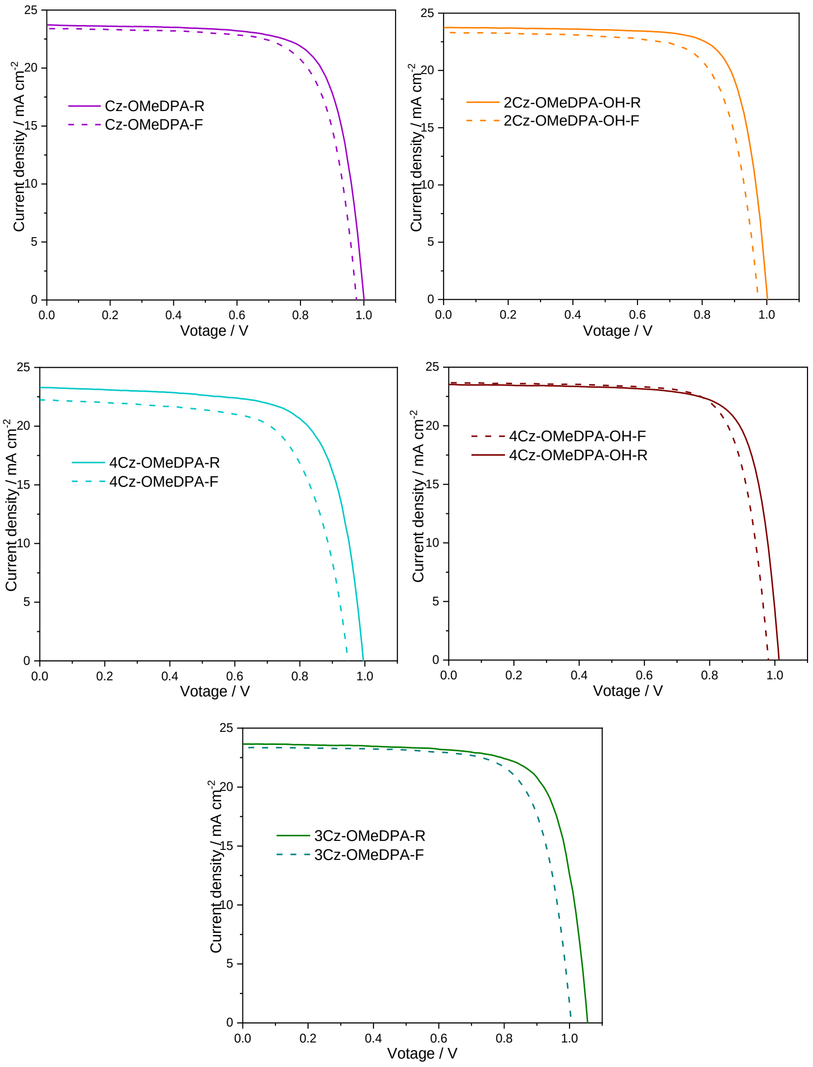

Figure S9. *J-V* hysteresis of the **Cz–OMeDPA, 2Cz–OMeDPA–OH, 4Cz–OMeDPA, 4Cz–OMeDPA–OH and 3Cz–OMeDPA.**

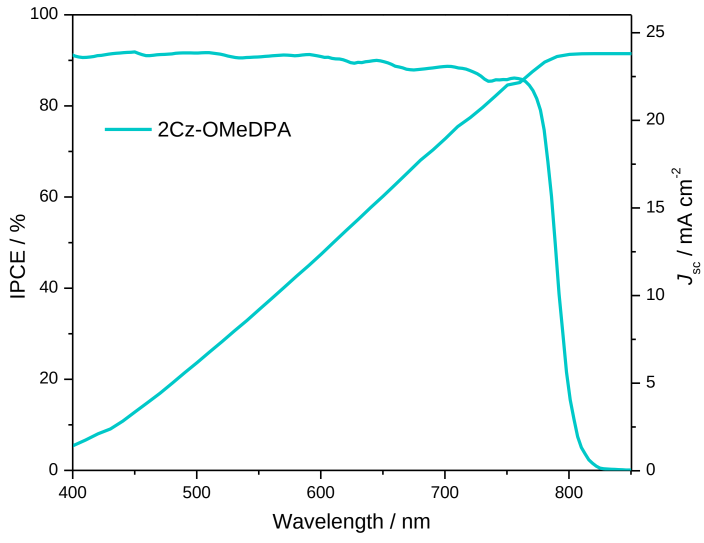

Figure S10. The IPCE of devices based on HTM of **2Cz-OMeDPA.**

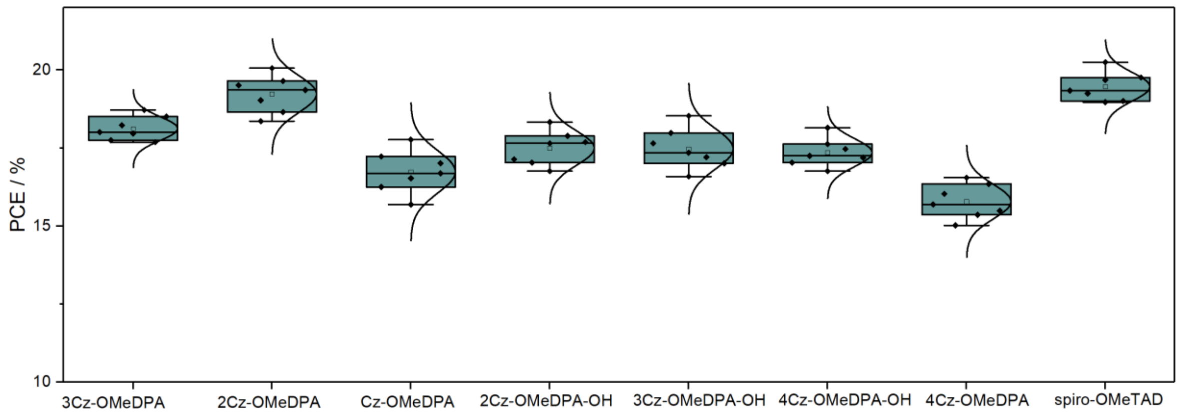

Figure S11. The statistic PCE of devices based on various HTMs.

References

[1] B. Xu, E. Sheibani, P. Liu, J. Zhang, H. Tian, N. Vlachopoulos, G. Boschloo, L. Kloo, A. Hagfeldt, L. Sun, *Adv. Mater.* 2014, 26, 6629–6634.
